# Gut Mycobiome Changes During COVID-19 Disease

**DOI:** 10.3390/jof11030194

**Published:** 2025-03-03

**Authors:** Danil V. Krivonos, Dmitry E. Fedorov, Ksenia M. Klimina, Vladimir A. Veselovsky, Svetlana N. Kovalchuk, Alexander V. Pavlenko, Oleg O. Yanushevich, Dmitry N. Andreev, Filipp S. Sokolov, Aleksey K. Fomenko, Mikhail K. Devkota, Nikolai G. Andreev, Andrey V. Zaborovsky, Sergei V. Tsaregorodtsev, Vladimir V. Evdokimov, Natella I. Krikheli, Petr A. Bely, Oleg V. Levchenko, Igor V. Maev, Vadim M. Govorun, Elena N. Ilina

**Affiliations:** 1 Research Institute for Systems Biology and Medicine (RISBM), 18, Nauchniy Proezd, 117246 Moscow, Russia; 2 Department of Molecular and Translational Medicine, Moscow Institute of Physics and Technology, State University, 141700 Dolgoprudny, Russia; 3 Lopukhin Federal Research and Clinical Center of Physical-Chemical Medicine of Federal Medical Biological Agency, ul. Malaya Pirogovskaya, 1s3, 119435 Moscow, Russia; 4 Federal State Budgetary Educational Institution of Higher Education “Russian University of Medicine” of the Ministry of Health of the Russian Federation, 127006 Moscow, Russiaphlppsokolov@gmail.com (F.S.S.); docfomenko@yandex.ru (A.K.F.); sv-tsaregorodtsev@ya.ru (S.V.T.);

**Keywords:** fungal community, COVID-19, gut microbiome

## Abstract

The majority of metagenomic studies are based on the study of bacterial biota. At the same time, the COVID-19 pandemic has prompted interest in the study of both individual fungal pathogens and fungal communities (i.e., the mycobiome) as a whole. Here, in this work, we investigated the human gut mycobiome during COVID-19. Stool samples were collected from patients at two time points: at the time of admission to the hospital (the first time point) and at the time of discharge from the hospital (the second time point). The results of this study revealed that *Geotrichum* sp. is more represented in a group of patients with COVID-19. Therefore, *Geotrichum* sp. is elevated in patients at the time of admission to the hospital and underestimated at the time of discharge. Additionally, the influence of factors associated with the diversity of fungal gut microbiota was separately studied, including disease severity and age factors.

## 1. Introduction

The human microbiota is a complex and heterogeneous system consisting of a variety of organisms. Modern metagenomics has made significant studies in the structure of the bacterial component of the human microbiota. However, there is a notable lack of attention directed towards variations in the fungal component of the microbiota. At the same time, there has been an increase in the number of cases of yeast-like and mold fungi acting as infectious agents of a nosocomial nature, causing severe generalized mycoses and skin lesions. The cases most often observed are those of yeast-like fungi of the genus *Candida* [1] and mold fungi of the genus *Aspergillus* [2]. The mycoses caused by this genus of fungi are difficult to treat in some cases.

The human gut microbiota is the most representative human biotope, reflecting the maximum variability of microorganisms that inhabit it. In addition to bacteria, fungi, archaea, and viruses, it includes a multitude of other microbial species. There is a growing trend in research on the gut microbiota to shift the focus towards the study of the rare biosphere, particularly the fungal component, the mycobiome [3,4]. In particular, ref. [4] identifies the most represented genera of fungal microorganisms in the human intestine, namely *Saccharomyces*, *Malassezia*, *Candida*, *Cyberlindnera*, *Penicillium*, *Cladosporium*, *Aspergillus*, *Debaryomyces*, *Pichia*, *Clavispora*, and *Galactomyces*. Furthermore, it describes characteristic changes in their relationships in different pathologies.

The gut mycobiome is typically distinctive across individuals in different geographical locations. Consequently, the joint analysis of external gut mycobiome sequencing data obtained from different countries led to the identification of four different fungal enterotypes [5]. The following enterotypes are distinguished based on the representation of the dominant genus: Sacc_type (*Saccharomyces* sp. dominated), Can_type (*Candida* sp. dominated), Asp_type (*Aspergillus* sp. dominated), and Asc_type (unclassified *Ascomycota* phylum dominated). However, it should be noted that this work does not include samples from Russia, which is also a valuable source of information in the context of studying changes in the fungal microbiota.

The gut biotope demonstrates the greatest variability of the human microbiome and serves as a natural reservoir of microorganisms that are functionally significant for human health. The field of metagenomics has revealed a close relationship between the mycobiome and the bacteriome, which can be described as a single microbial community. Furthermore, although the human gut mycobiome is distinguished by a relatively low diversity compared to the bacteriome, alterations in its qualitative and quantitative composition are even more pronounced in the context of various pathologies, such as inflammatory bowel diseases [4]. The high prevalence of several fungal species identified by metagenomic studies suggests that a limited set of fungal species may exist in the human intestine, defining the core mycobiota. However, a consensus has yet to be reached in defining the set of fungal species characteristic of the mycobiome of a healthy individual. This limitation makes it challenging to interpret the detection of potential fungal pathogens in clinical samples from patients receiving hospital care.

Interest in the mycobiome has especially grown against the background of the COVID-19 pandemic. An increased representation and change in the spectrum of fungal microorganisms was observed in patients with COVID-19 [6]. Complications from fungal infections were a significant cause of mortality among patients [7,8]. At the same time, there is no understanding of the patterns of formation of a complicated picture of the course of COVID-19 through the addition of nosocomial bacterial or fungal biota. Furthermore, the literature also describes that the presence of certain fungal taxa is determined by the type of human diet [4].

A recent study [9] compared the gut and oral mycobiomes of patients with confirmed COVID-19 after recovery and a year later. The Simpson index revealed that the alpha diversity of the gut mycobiome was lowest in the control group and greatest in patients immediately after discharge. After a year, the alpha diversity of the gut mycobiome decreased and approached the value observed in the control group.

Although a study has been conducted to examine alterations in the mycobiome following recovery from COVID-19, the question of variations in fungal mycobiota during the course of the disease itself remains unsolved. In this study, we sought to assess changes in the mycobiota resulting from the effects of the inpatient therapy of COVID-19 and also expand the geographic range of mycobiome characteristics to samples from Russia.

## 2. Materials and Methods

### 2.1. Sample Collection

The collection was made up of fecal samples taken from 81 patients with COVID-19. Fecal samples from sick people were collected at two time points: 65 were collected at the time of hospital admission, and 54 at the time of discharge. Samples from patients were collected in the Clinical Medical Center “Kuskovo” from April to June 2021. All the patients signed informed consent to participate in the study. The study did not include patients diagnosed with cancer.

Stool samples were collected in sterile containers in a sample volume of 5 to 15 mL. The samples were stored at −70 °C.

Patient samples were collected at two distinct time points: the initial collection occurred at the time of admission to the hospital (the first time point), while the second collection was conducted at the time of discharge (the second time point).

### 2.2. ITS Sequencing

#### 2.2.1. DNA Isolation

The total DNA was extracted from all fecal samples (*n* = 102) using the MagMAX™ Microbiome Ultra Nucleic Acid Isolation Kit, with bead tubes and the KingFisher™ Purification System (Thermo Fisher Scientific, Waltham, MA, USA), in accordance with the instructions provided by the manufacturer. The DNA was subsequently quantified on a Qubit 4 fluorometer by the Quant-iT dsDNA BR Assay Kit (Thermo Fisher Scientific, Waltham, MA, USA).

#### 2.2.2. NGS Library Preparation

The variable regions ITS1-5.8S-ITS2 were amplified using the primers ITS5 (GGAAGTAAAAGTCGTAACAAGG) and ITS4 (TCCTCCGCTTATTGATATGC) [10] and the Tersus PCR kit (Evrogen, Russia). Samples were barcoded using unique combinations of indexing primers, which are analogs of primers from the Nextera XT Index kit v2. The sequencing procedure was conducted using the MiSeq instrument and the MiSeq Reagent Kit v3 (600-cycle) consumable kit (Illumina, San Diego, CA, USA).

### 2.3. Bioinformatic Analysis

Adapter read sequences were trimmed using Trimmomatic v0.39 [11]. Primer sequences were trimmed via cutadapt v4.6 [12]. Amplicon Sequence Variants (ASVs) were constructed using the dada2 v1.30.0 software package [13]. Reads were filtered using the filterAndTrim function with the following parameters (truncLen = c(150,150), maxN = 0, maxEE = c(2,3), truncQ = 2, compress = TRUE, multithread = TRUE). The denoising of forward and reverse reads was conducted separately. The mergePairs function was employed for merging with the justConcatenate parameter, as the sum of reads did not permit comprehensive coverage of the amplicon region of the full-length ITS cluster. Thus, each ASV sequence contained 10 N letters in the center of the sequence. The chimera removal stage was carried out using removeBimeraDenovo (method = “consensus”). Only those samples with a sum of taxon counts exceeding 100 were subjected to further analysis.

dada2’s built-in assignTaxonomy function resolved fungal taxonomy incorrectly [14]. So, the taxonomy assignment was conducted by mapping against the UNITE database (unite_04.04.2024) [15] using blastn. A cutoff of 80% for query coverage and an E-value of 1 × 10^−30^ were employed. To elucidate the taxonomy of individual sequences, mapping of ASV sequences to the nt database using blastn was used.

Samples in which the total number of numbers for all OTUs was less than 10 were discarded. ASV sequences were subjected to additional post-clustering curation using LULU v0.1.0 [16]. A matchlist for LULU was produced with VSEARCH vv2.27.0 [17]. The analysis scheme is visualized in Supplementary S1 Figure S1.

The statistical analysis and data visualization were conducted using the R and python 3 software. The differential abundance analysis was conducted using the DESeq2 v1.42.1 [18] software. The DESeq2 algorithm was employed with a Wald test, and the sfType parameter was set to poscounts. The search for fungal enterotypes was conducted using Dirichlet Multinomial Mixtures (DMMs) [19] and the AIC, BIC, and Laplace metrics. To analyze associations, we used the nonparametric PERMANOVA test (with the Atchenson distance, 999 iterations) and additional testing using PERMDISP (with the Atchenson distance, 999 iterations).

The FunFun tool was used for the purpose of annotating gene content [20]. Differential representation of individual orthology groups was achieved through the calculation of log2FoldChange functions between groups. As a statistical test for the purpose of comparing the distributions of functions within the group, the Mann–Whitney U-test with Benjamin Hochbeck correction was used. Cutoff criteria for differentially represented features were *p*-value < 0.05 and −2 ≤ log2FoldeChange ≤ 2.

A multiple alignment for the assembled ASVs was created with MAFFT [21], and a phylogenetic tree was obtained via FastTree [22] (Maximum Likelihood approach with 1000 bootstrap iterations). The phylogenetic tree visualization was obtained via iTOLL.

## 3. Results

In total, the 102 fecal samples recovered from 81 COVID-19 patients, ranging in age from 25 to 81 years old, were analyzed using the ITS-amplicon metagenomic sequencing. Of them, 65 samples were collected at the time of hospital admission, and 54 at the time of discharge.

### 3.1. ASV Calling Results and Decontamination

The construction of ASVs using dada2 resulted in the acquisition of 3749 unique sequences. Following the determination of taxonomy with the selected parameters, 360 ASVs were identified as fungal ITS. After decontamination via Decontam, 10 ASV sequences were identified as contaminants. In the final stage of agglomeration with LULU, 178 ASVs were obtained. The assembled ASV sequences are included in Supplementary S2. As a result of the entire data processing, 102 samples corresponding to 76 people were retained. Therefore, the array comprised 61 samples corresponding to the first time point (moment of admission to the hospital) and 41 samples corresponding to the second time point (the moment of discharge from the hospital).

### 3.2. Taxonomic Composition Description

**Figure 1:**
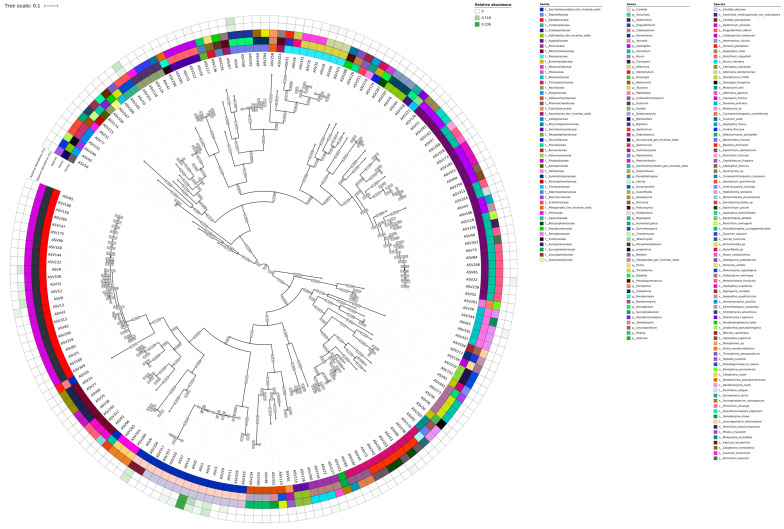
Phylogenetic tree for assembled ASVs. Bootstrap support visualized with light blue color. Mean relative abundance was illustrated for each group separately (F1—1st time point; F2—2nd time point). The tree was rooted in the midpoint.

A phylogenetic tree was constructed for all collected ASV sequences that did not undergo agglomeration (Supplementary S1 Figure S2). The resulting tree demonstrates notable divergence among taxa of the same species. To minimize divergence between taxonomic annotations of phylogenetically similar sequences, the ASV sequences were subjected to the agglomeration procedure using LULU. A phylogenetic tree was also constructed for the sequences after agglomeration, as visualized in Figure 1.

The majority of the identified fungal species belong to the genera *Candida*, *Geotrichum*, *Engyodontium*, and *Ascochyta*. The genus *Geotrichum* is represented exclusively by the species *Geotrichum silvicola*. Similarly, the genus *Candida* is represented by the species *Candida albicans* and *Candida parapsilosis*. The genus *Engyodontium* comprises a single species, *Engyodontium album*, which is represented by two distinct sequences, ASV10 and ASV183. The genus *Ascochyta* is represented by a single species, *Ascochyta medicaginicola*, which is further characterized by three sequences: ASV292, ASV340, and ASV4.

### 3.3. Association of Mycobiome with Patient Comorbidity and Treatment Procedure

**Figure 2:**
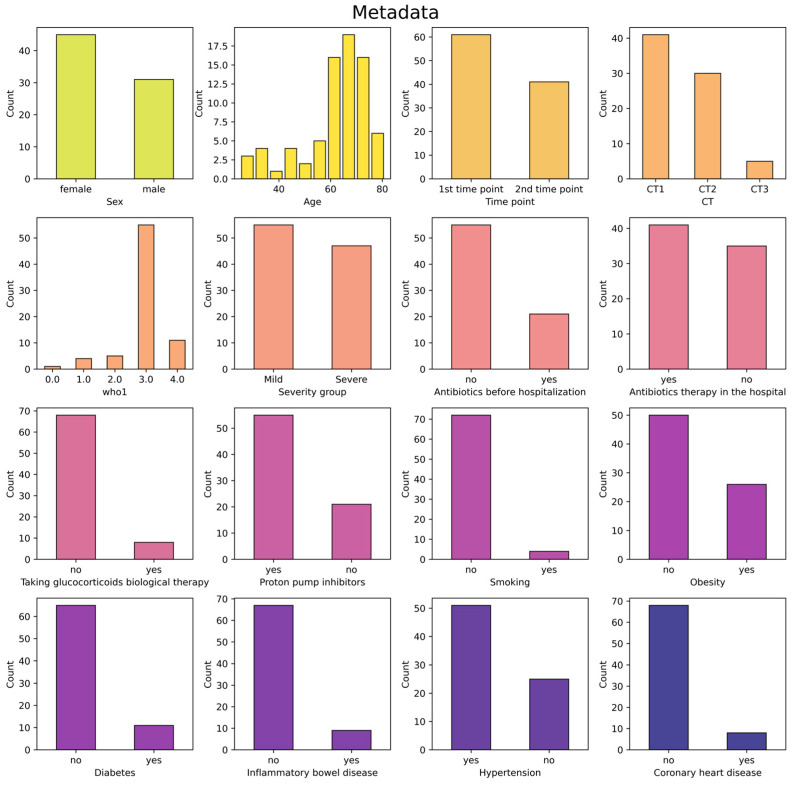
Presentation of metadata for patients. CT is computed tomography score (CT1—less than 25–50% of the lungs are affected; CT2—moderate pneumonia, 25–50% of the lungs are affected; CT3—50–75% of the lungs are affected; CT4 is a severe form of pneumonia, affecting >75%). The who1 score is the WHO performance score at admission to the hospital. The severity group is a combined score of disease severity according to CT and the WHO performance score scale.

The severity of the disease was evaluated based on computed tomography (CT) data and classified into four categories according to the percentage of lung damage: CT1—less than 25–50% of the lungs are affected; CT2—moderate pneumonia, 25–50% of the lungs are affected; CT3—50–75% of the lungs are affected; CT4 is a severe form of pneumonia, affecting >75% of the lungs [23]. Additionally, the severity of the disease was evaluated according to the World Health Organization performance scores (WHO-PSs) [24], in accordance with the hospital’s internal regulations (further WHO1). To combine the data on the severity of the disease assessed by CT and the WHO-PS scale, we employed the scheme previously published in the work [25] (next severity group). The visualization of the division of samples into groups is presented in Supplementary S1 Figure S7. All metadata for the samples was visualized in Figure 2.

**Figure 3:**
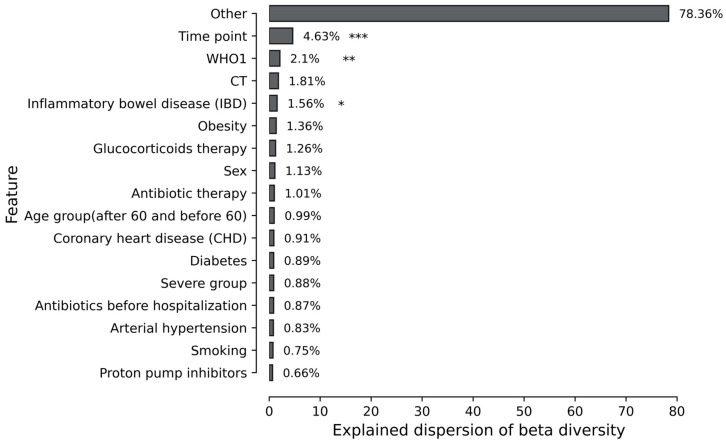
PERMANOVA results for different patient factors (*—*p*-value ≤ 0.05, **—*p*-value ≤ 0.01, ***—*p*-value ≤ 0.001). CT is computed tomography score (CT1—less than 25–50% of the lungs are affected; CT2—moderate pneumonia, 25–50% of the lungs are affected; CT3—50–75% of the lungs are affected; CT4 is a severe form of pneumonia, affecting >75%). The who1 score is the WHO performance score at admission to the hospital. The severity group is a combined score of disease severity according to CT and the WHO scale.

To identify covariates associated with the mycobiome, we used the nonparametric PERMANOVA test (with the Atchenson distance, 999 iterations) with additional verification of the results obtained using PERMDISP (with the Atchenson distance, 999 iterations). The results obtained are combined into a single table in Supplementary S1 Table S1. The percent of explained dispersion of beta diversity from PERMANOVA results is illustrated in Figure 3.

The PERMANOVA analysis revealed about 21.64% of the observed variations in beta diversity were attributable to methodological factors. The statistical analysis revealed that three factors were significantly associated with mycobiome beta diversity: time point value, WHO1 index, and the presence of inflammatory bowel disease (IBD). The PERMDISP test revealed that the observed statistically significant differences in time points and WHO1 are attributable to either distinct variance. However, IBD has no statistical significance according to PERMDISP, which suggests that the obtained results are not a consequence of differences in the variance between the groups. Nevertheless, it is noted that the analyzed sample size is too small to provide sufficient confidence in the obtained results for patients with confirmed IBD.

A closer examination of the impact of IBD on alpha diversity revealed that samples from patients with confirmed IBD exhibited higher fungal alpha diversity than samples from patients without IBD (Supplementary S1 Figure S8). The distributions of alpha diversity values were found to be statistically significant (*p*-value < 0.05).

### 3.4. Mycobiome Differences Across Time Points

#### 3.4.1. Alpha and Beta Diversity Estimation for Different Time Points

**Figure 4:**
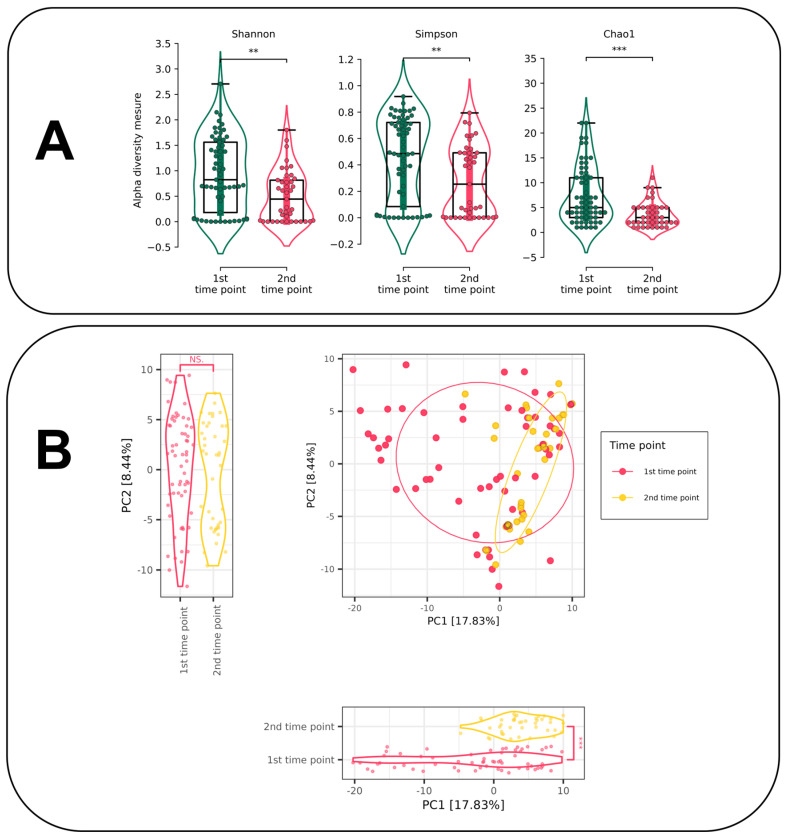
(**A**) Alpha diversity for different time points for ASV level (Mann–Whitney U-test results: **—*p*-value ≤ 0.01, ***—*p*-value ≤ 0.001). (**B**) PCoA plot with Atchenson distance for samples from different time points (NS.—*p*-value > 0.05, ***—*p*-value ≤ 0.001).

Alpha diversity was assessed using three measures: Shannon, Simpson and Chao1. Alpha diversity is significantly higher at the first time point (the moment the patient is admitted to hospital) than at the second time point (the moment of discharge from hospital). Statistical significance was assessed using the non-parametric Mann–Whitney U test. Statistically significant differences were observed when assessing alpha diversity using all three measures (*p*-value < 0.05). At the same time, a similar trend towards underestimation of alpha diversity at the time of discharge was observed at the ASV level (Figure 4A) and at the species level (Supplementary S1 Figure S12).

The decomposition of mycobiome samples was conducted using Principal Coordinates Analysis (PCoA) with Euclidean distance and clr transformation of the original data (the so-called Atchinson distance) (Figure 4B). The statistical differences between samples by components were determined using the Mann–Whitney U-test. A similar decomposition was performed for data with the Bray–Curtis dissimilarity (Supplementary S1 Figure S4).

The decomposition does not reveal any discernible clustering between the various time points. However, the samples exhibit statistically significant differences in the first PCoA component (*p*-value < 0.05). A similar statistically significant difference is also observed in the second PCoA component of the decomposition obtained using the Bray–Curtis dissimilarity (Supplementary S1 Figure S4). To identify fungal enterotypes, sample clustering was performed using DMM, but this search did not reveal any clustering (Supplementary S1 Figure S5).

#### 3.4.2. Differential Abundance Analysis for Different Time Points

**Figure 5:**
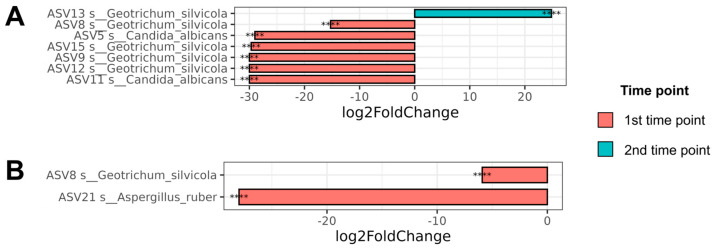
Results of differential abundance analysis obtained via DESeq2. (**A**) ASV level; (**B**) species level (****—*p*-value ≤ 0.0001).

The differential representation between samples at different time points was evaluated using DESeq2. At the level of individual ASVs, ASV13 *Geotrichum silvicola* was differentially represented at the second time point (prevalence = 0.04). At the first time point, ASV8 *Geotrichum silvicola* (prevalence = 0.24), ASV5 Candida albicans (prevalence = 0.13), ASV15 *Geotrichum silvicola* (prevalence = 0.18), ASV11 Candida albicans (prevalence = 0.21), ASV9 *Geotrichum silvicola* (prevalence = 0.19), and ASV12 *Geotrichum silvicola* (prevalence = 0.05) were overrepresented. DESeq2 results for ASV level are visualized in Figure 5.

At the species level, representation is observed only at the first time point for the sequences of *Geotrichum silvicola* (prevalence = 0.59) and Aspergillus ruber (prevalence = 0.02). The DESeq2 results for the species level are presented in Figure 5.

### 3.5. Severity as a Factor of Fungal Alpha Diversity

**Figure 6:**
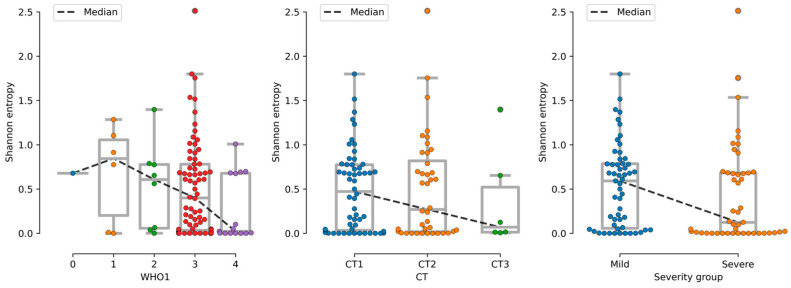
Changes in the trend of alpha diversity depending on the severity of the disease (species level). CT is computed tomography score (CT1—less than 25–50% of the lungs are affected; CT2—moderate pneumonia, 25–50% of the lungs are affected; CT3—50–75% of the lungs are affected; CT4 is a severe form of pneumonia, affecting >75%). The who1 score is the WHO performance score at admission to the hospital. The severity group is a combined score of disease severity according to CT and the WHO scale.

The assessment of alpha diversity change by groups revealed no statistically significant differences (*p*-value > 0.05). However, the distribution medians tend to be lower in groups with a more severe course of the disease. A similar trend is observed for all three disease severity levels, particularly at the species level (Figure 6). A weaker trend is noted at the ASV level (Supplementary S1 Figure S11). The most striking trend towards a decrease in alpha diversity is observed when assessing the level of lung damage using CT. Therefore, species alpha diversity and ASV alpha diversity tend to decrease with an increasing degree of lung damage.

Following the assessment of differential representation between the mild and severe groups using DESeq2, no differentially represented sequences were identified at the ASV level. Conversely, the species *Apiotrichum domesticum* (log2FC = −25.52, *p*-value = 1.85 × 10^−18^, prevalence = 0.04) exhibited a tendency towards overrepresentation in the severity group samples. The prevalence of *Apiotrichum domesticum* is sufficiently low to qualify as a significant result.

### 3.6. Age-Related Mycobiome Associations

**Figure 7:**
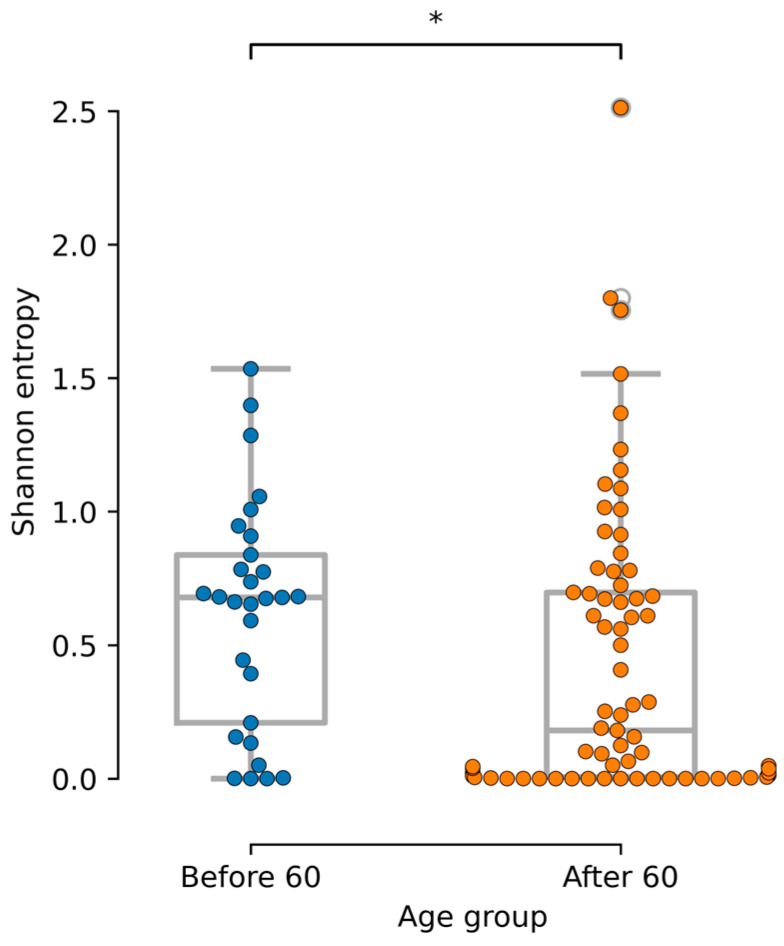
Changes in the trend of alpha diversity depending on age group (*—*p*-value < 0.05).

The alpha diversity of the gut mycobiome for groups comprising individuals under the age of 60 was found to be significantly higher than that of the cohort comprising individuals aged 60 and above (*p*-value < 0.05). The results are represented graphically in Figure 7. In the differential abundance estimation results for the group of people over 60, the sequences of ASV13 *Geotrichum silvicola* (log2FC = 23.012, *p*-value = 9.36 × 10^−13^, prevalence = 0.04) and ASV12 *Geotrichum silvicola* (log2FC = 27.63, *p*-value = 9.51 × 10^−18^, prevalence = 0.05) are also overrepresented. At the species level, no significant differences in the representation of fungi were observed between age groups.

### 3.7. Functional Annotation via FunFun

The FunFun annotation of the obtained sequences did not reveal any differentially represented biosynthetic pathways between different time points and between the severe groups (mild and severe). However, no differentially represented orthology groups were identified.

The search for the most variable functions was conducted using the variation coefficient and filtering by the prevalence of the function (prevalence > 0.5 and variation coefficient > 1). Among such functions, FunFun prediction artifacts were still preserved. However, upon detailed consideration of the obtained results, the pathways of biosynthesis of secondary metabolites were noted. Nevertheless, the obtained results require additional experimental confirmation.

## 4. Discussion

The most prevalent fungal genus *Candida* is represented by two species: *Candida albicans* was identified in 79/102, 77% of samples, and *Candida parapsilosis* was found in 41/102, 40%. The presence of *C. parapsilosis* and *C. albicans* in gut microbiomes has been repeatedly reported, and they are recognized as opportunistic pathogens and components of the normal human microbiota [3,26]. Considering that no different enterotypes were identified in the analyzed array with a prevalence of *Candida* spp., we suggest that the mycobiomes largely belong to the Can_type enterotype, which previously was described in the literature [5].

The second most common genus in our dataset is *Geotrichum*. Sequences representing the genus *Geotrichum* are predominantly annotated as *Geotrichum silvicola* found in 38/102, 37% of the samples. However, with a detailed analysis of sequences annotated as *Geotrichum silvicola*, ambiguity arises with the exact identification of the *Geotrichum* species since the closest sequence from the UNITE database (KX218268) has great similarity to both *Geotrichum silvicola* and *Geotrichum candidum* when aligned to nt. *G. silvicola* is a well-characterized organism that has been repeatedly detected in human fecal samples in numerous studies [27,28,29]. In early publications, species of the genus *Geotrichum*, including *Geotrichum candidum*, were observed in various samples such as soil, plant, and insect samples [28,29]. This indicates a rather wide range of habitats for this fungus. It is quite possible that colonization by this fungus has already occurred in the clinic through contact with contaminated material. However, the genus *Geotrichum* has also been observed in gut mycobiome samples and can be the reason for geotrichosis disease [28].

*Engyodontium album* is present in 26/102, 25% of samples, and is also described as a pathogen of cattle and humans [30,31]. However, it has not previously been described in the context of human gut microbiota. *E. album* is a relatively common environmental organism, frequently encountered on surfaces such as paper, jute, and linen [32]. *Cladosporium herbarum* is present in 23/102 samples, approximately 23% of all samples, and is also one of the most frequently detected fungi in the gut mycobiome [33].

Previously published works did not describe some of the fungi we found in the data as normal components of the human microbiota. For instance, *Ascochyta medicaginicola* is present in 15/102, 15% of our set of samples. These fungi are plant pathogens, and it appears that they may enter the human intestine with food or external pollen [34]. A similar situation occurs with *Penicillium roqueforti*. *P. roqueforti* was identified in 16/102, 16% of the samples. In the literature, other species of the *Penicillium* genus have also been repeatedly observed in samples of human gut microbiota [27], but *P. roqueforti* is actively utilized in industrial cheese production technology and is often found in food products. Therefore, its colonization can have both a temporary effect and a permanent role within the human microbial community.

Further, we focus on changes in gut mycobiome composition that happened between the two time points corresponding to patients’ admission to the hospital and discharge from it. The results of PCoA data decomposition revealed significant discrepancies between the samples at different time points, as indicated by PC1 (Figure 4B). Consequently, there are evident indications that differentiate the mycobiome state at distinct time points. A comparison of the two time points revealed a lower alpha diversity at the second time point relative to the first one by all applied metrics (Figure 4A).

The state at discharge gradually approaches that of healthy biota, and the decrease in alpha diversity in patients at discharge is also consistent with this. This is also consistent with the literature, which describes that patients immediately after recovery from COVID-19 have a higher alpha diversity of the gut mycobiome than the same patients one year after recovery [9]. It is probable that this reduction in diversity is attributable to the inability of certain fungi to occupy their ecological niche. The fungi that emerged at the initial time point may have been transient, and due to their inability to adapt to the conditions of the human intestine, they were likely eliminated from the system by the time of discharge.

In our study, the greatest fungal diversity is observed at the initial time point, which corresponds to the patient’s admission to the hospital, and the onset of the disease and depression of immunity. This may be a consequence of both the individual characteristics of the fungus and its gradual displacement by the human immune system or bacterial biota. The mycobiota is typically opportunistic and develops precisely when immunity is compromised. In particular, this has been demonstrated previously for fungi of the genus *Candida* [35,36,37,38]. While research has focused on Candida albicans, it can be inferred that analogous mechanisms of interaction with the immune system may exist for other fungal species. However, in order to be able to answer this question with greater certainty, further research is needed on the mycobiota and the interactions between the human immune system and individual types of fungi.

At the same time, the differential abundance analysis revealed that *Geotrichum silvicola* and *Aspergillus ruber* were overrepresented in patient samples at the first time point. *Aspergillus ruber* was found in only 9/102, 2% of samples, which is too little to clearly indicate any significant underrepresentation in different groups. At the same time, *Geotrichum silvicola* was significantly more common in the samples, which gives greater confidence in the result obtained. The decrease in the relative abundance of *Geotrichum silvicola* at the second time point is apparently due to its response to the immune state. Similarly, as we assume, the weakening of the human immune system at the first time point, which actually corresponds to the acute phase of the disease, apparently affected the growth of individual representatives of the fungal community. However, it should be noted that this is only an assumption, and the interaction of *Geotrichum silvicola* with the human immune system has yet to be studied. In nature, *Geotrichum silvicola* is a saprophyte and is not a direct human pathogen [3,27]. However, other fungi of the genus *Geotrichum* have been described as human opportunists, which may indirectly indicate that *G. silvicola* may also play an opportunistic role in the intestinal microflora [39,40].

Among the factors associated with beta diversity, it was observed that the presence of inflammatory bowel disease (IBD) in a patient exerts an influence on the state of the mycobiome. A limitation of the present study is the small number of patients with confirmed IBD in the analyzed group, which precludes a reliable investigation of this phenomenon. It is important to note, however, that the available data already indicate that the alpha diversity of the gut mycobiome for patients with IBD is higher than that of patients without IBD. Nevertheless, the bacterial biota is also characterized by a relatively low alpha diversity of gut bacterial biota for patients with IBD [41,42] compared to individuals without IBD. It should be noted that the association of IBD with the mycobiota requires further investigation with an expanded group of patients with IBD.

It is also noteworthy that antibiotics exerted no discernible impact on beta and alpha diversity (*p*-value > 0.05). The initial assumption was that the action of antibiotics would result in a decrease or change in the bacterial biota and an increase in fungal diversity. Furthermore, this increase in fungal alpha diversity following antibiotic therapy has been previously demonstrated [43]. However, no effects of antibiotics were observed in our array. Similarly, it is noteworthy that proton pump inhibitors also had no effect on the gut mycobiota. Nevertheless, it is possible that the effect of individual drugs on the mycobiota may be significant and requires future investigation. In addition, the sample included a relatively small number of patients with IBD, which complicates the determination of a clear association between IBD and increased fungal alpha diversity.

Disease severity was assessed using computed tomography (CT) scans, the international WHO-PS measure, and a combined assessment of these two parameters. In this study, we also found that fungal alpha diversity tended to decrease with increasing disease severity. This result was reproduced for all three measures of disease severity, with the trend toward decreasing alpha diversity with increasing disease severity being much more pronounced at the species level and less so at the ASV level. This may probably be due to the development of just one dominant fungus, which reduced the overall fungal diversity in general. However, the reasons for the observed phenomenon are yet to be reliably studied in the future. As a result of assessing the differential representation of fungi, no reliable results were obtained with a relatively high prevalence in the array.

Due to the somewhat biased age sample, the present study divided the samples into two age groups: under 60 and over 60 years. A large number of publications describe serious changes in human metabolism at 44 and 60 years of age [44,45]. Our data were fairly balanced by age to examine change in people under 44 but could be subdivided into groups under and over 60 years of age. The fungal alpha diversity also tends to differ between age groups (*p*-value < 0.05). In the analyzed array, a significant increase in fungal alpha diversity is observed in the group of people over 60, and the decrease in fungal diversity with age is also consistent with the literature data [3].

FunFun gene content annotation did not reveal any differentially represented groups between time points and severity groups (mild and severe). This could also be attributed to the limitations of the approach, the sensitivity or specificity of the array itself, or the relatively low prevalence of minor fungi in it.

## 5. Conclusions

The analysis demonstrated a decline in alpha diversity of the gut mycobiome at the time of hospital admission, as compared to the time of hospital discharge. Additionally, the proportion of relative representation of *Geotrichum* sp. exhibited a notable variation between time points, with an initial overrepresentation at the first time point and subsequent underrepresentation at the second.

Additionally, the severity of the disease is correlated with alpha diversity in the gut mycobiome, with a reduction in fungal diversity observed in patients with more severe disease. Additionally, it was observed that patients with IBD exhibited higher alpha diversity than those without IBD. However, further investigation is necessary to confirm this finding. Among age-related mycobiome associations, it was noted that patients over 60 years of age exhibited significantly lower fungal diversity, a finding that is also supported by literature data. Despite this work progress, there is still a significant number of questions about the formation of fungal communities and the so-called core component.

## Data Availability

All data obtained during the course of the research and subjected to the requisite preprocessing have been uploaded to the NCBI BioProject under the accession number PRJNA1193860. The R notebooks and Jupyter lab notebooks (python3 scripts) are available for download from GitHub via the following link: https://github.com/DanilKrivonos/Gut-mycobiome-changes-during-COVID-19-disease (accessed on 1 March 2025).

## Supplementary Materials

The following supporting information can be downloaded at: https://www.mdpi.com/article/10.3390/jof11030194/s1, Supplementary S1: additional visualization, Supplementary S2: list of assembled ASV.
