# Supplementary material for: Gut Mycobiome Changes During COVID-19 Disease
Source: J Fungi (Basel). 2025 Mar 3;11(3):194. doi: 10.3390/jof11030194 (PMC11943151; doi:10.3390/jof11030194)
Supplement: Supplementary file 1 [file jof-11-00194-s001.zip › Supplementary1.pdf]

# Supplementary 1

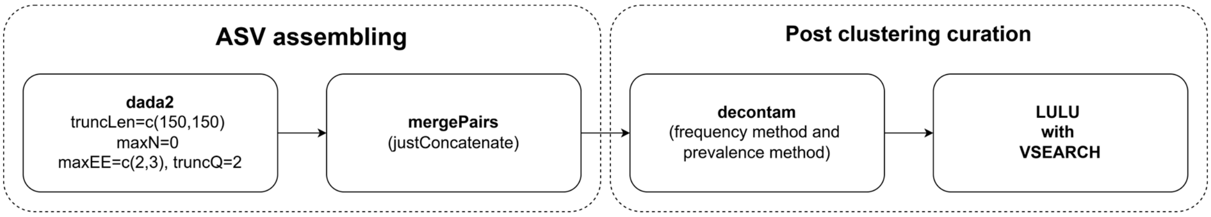


Figure S1 ASV assembling methods and ASV post processions.

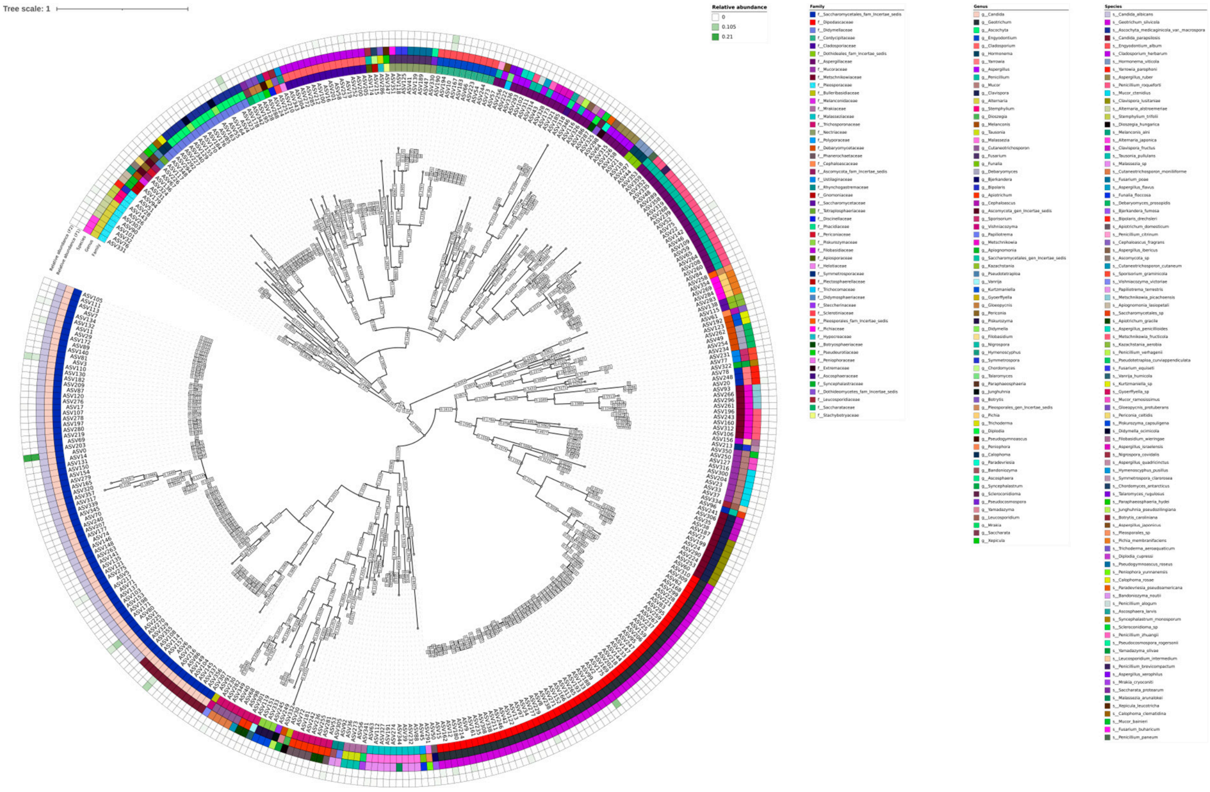


Figure S2 Phylogenetic tree for assembled ASV before decontamination and agglomeration. Bootstrap support visualized with light blue color. Mean relative abundance was illustrated for each group separately (healthy - for healthy cohort; F1 - infected cohort 1st time point; F2 - infected 2ed time point). Contamination ASV was marked by triangles. Tree was rooted in the midpoint.

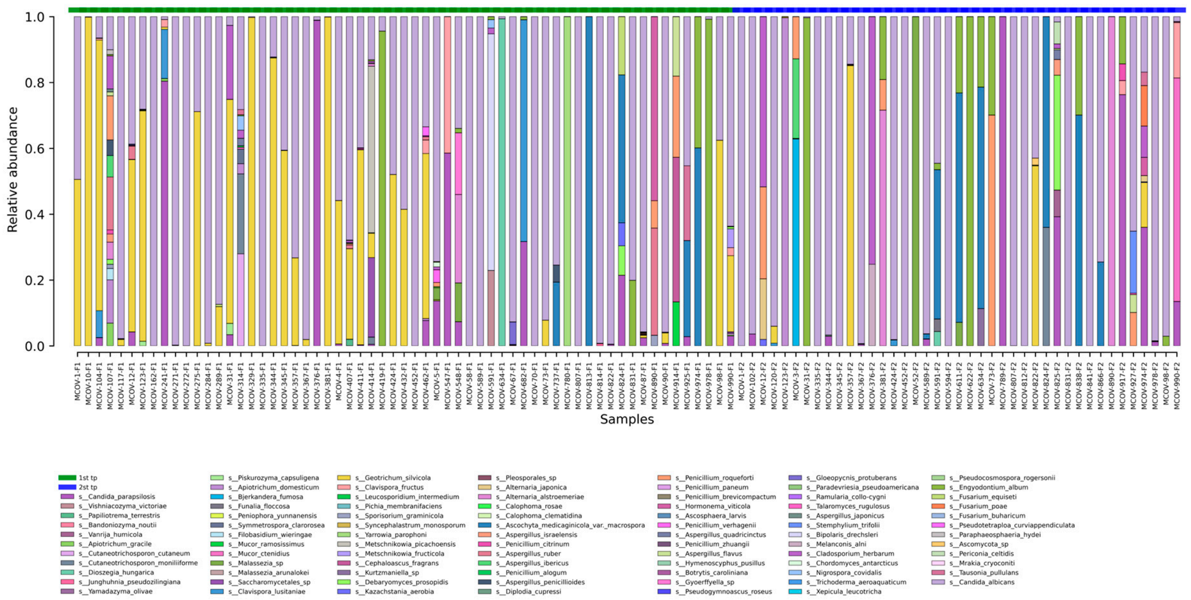


Figure S3 ASV assembling methods and ASV post processions.

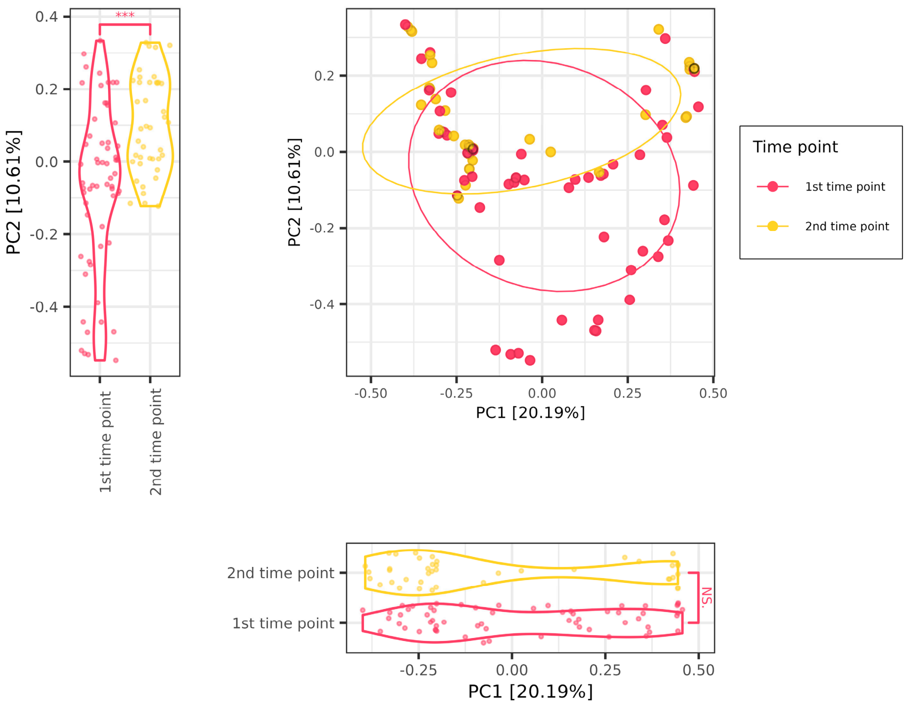


Figure S4 PCoA with Bray–Curtis distance decomposition for samples from different time points (NS. - p-value > 0.05, *** - p-value $\leq 0.001$).

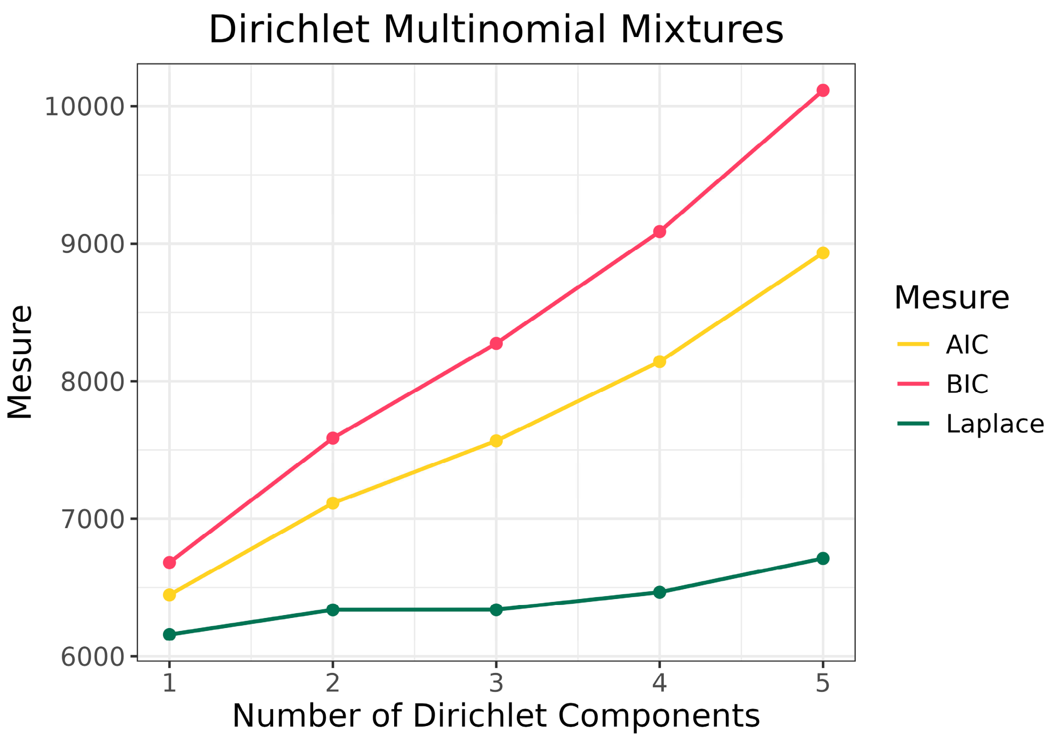


Figure S5 Dirichlet Multinomial Mixtures (DMM) mycobiome clustering. AIC - Akaike information criterion; BIC - Bayesian information criterion.

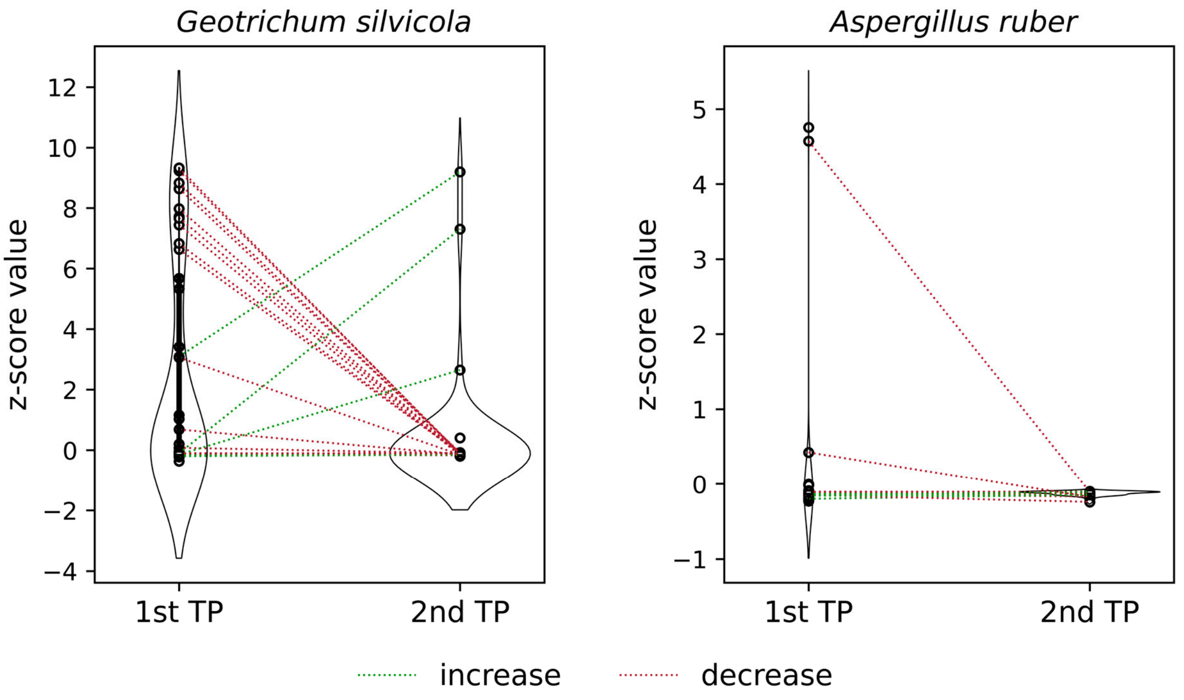


Figure S6 Z-score values for differentially represented fungal species between time points. For paired samples, changes are marked with dotted lines (red for increased species, green - for decreased species).

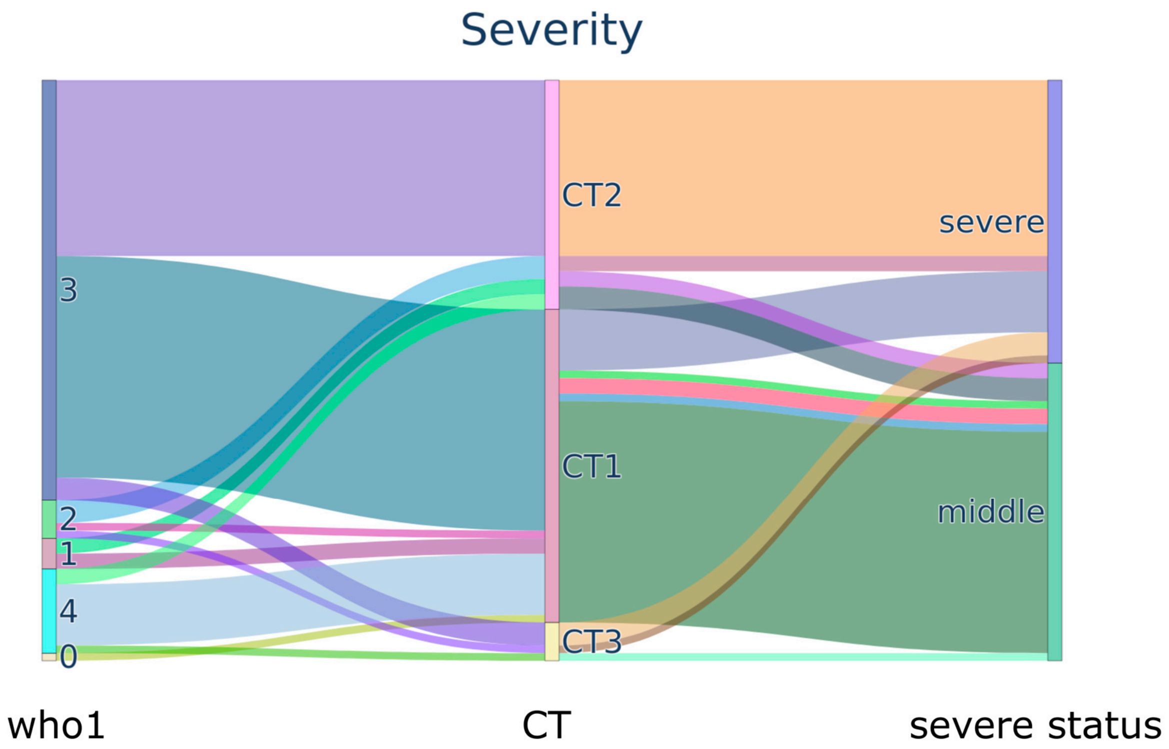


Figure S7 Results of dividing patients into middle and severe groups according to CT and WHO1 value. CT is computed tomography score (CT1 - less than 25-50% of the lungs are affected; CT2 - moderate pneumonia, 25-50% of the lungs are affected; CT3 - 50-75% of the lungs are affected; CT4 is a severe form of pneumonia, affecting >75%). who1 score is WHO performance scores at admission to the hospital. Severe group is a combined score of disease severity according to CT and the WHO scale.

Table S1. Results of the analysis of metadata associated with beta diversity.

| Factor | p-value (PERMANOVA) | p-value (PERMDISP) |
|---|---|---|
| CT | 0.509 | 0.565 |
| WHO1 (WHO performance scores at admission to the hospital) | **0.006** | **0.019** |
| Severe status (Mid or Severe) | 0.483 | 0.453 |
| Inflammatory bowel disease (IBD) | **0.040** | 0.453 |
| Arterial hypertension | 0.582 | 0.988 |
| Time point | **0.001** | **0.002** |
| Antibiotic therapy | 0.311 | 0.979 |
| Antibiotics before hospitalization | 0.500 | 0.52 |
| Smoking | 0.664 | 0.865 |
| Sex | 0.192 | 0.383 |
| Age group (after 60 and before 60) | 0.358 | 0.109 |
| Diabetes | 0.469 | **0.038** |
| Obesity | 0.082 | 0.92 |
| Coronary heart disease (CHD) | 0.480 | **0.015** |
| Glucocorticoids therapy | 0.129 | 0.197 |
| Proton pump inhibitors | 0.857 | 0.949 |

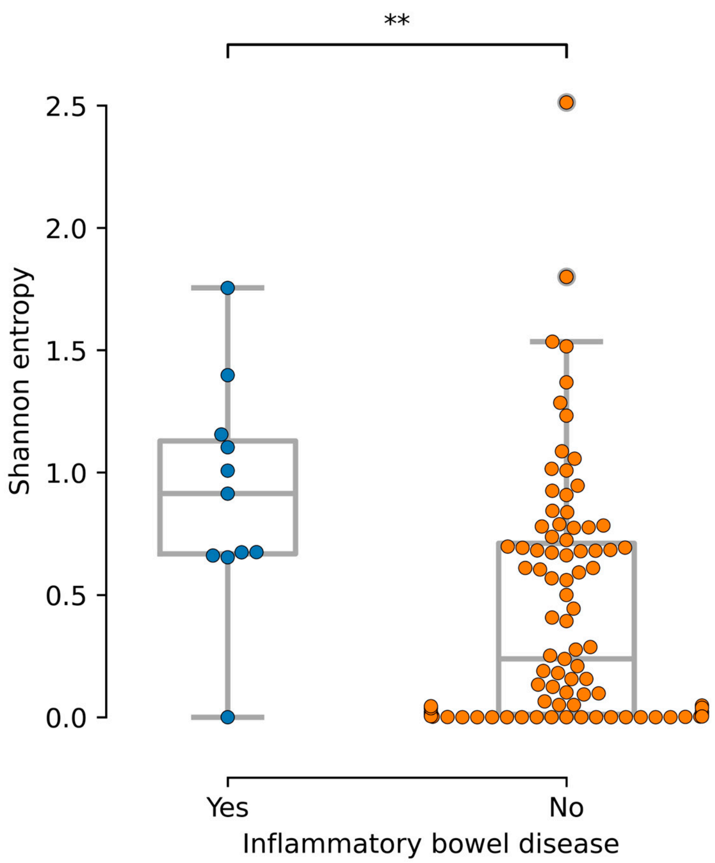


Figure S8 Changes in alpha diversity depending on the presence of IBD (Inflammatory bowel disease) in a patient.

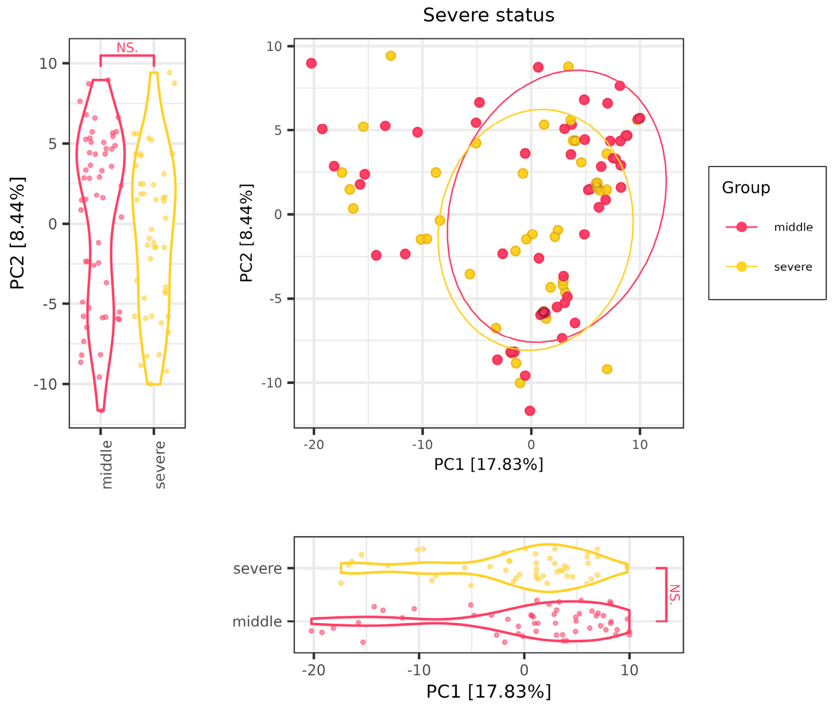


Figure S9 PCoA with Atcheson distance decomposition for samples from different severity groups (NS. - p-value > 0.05).

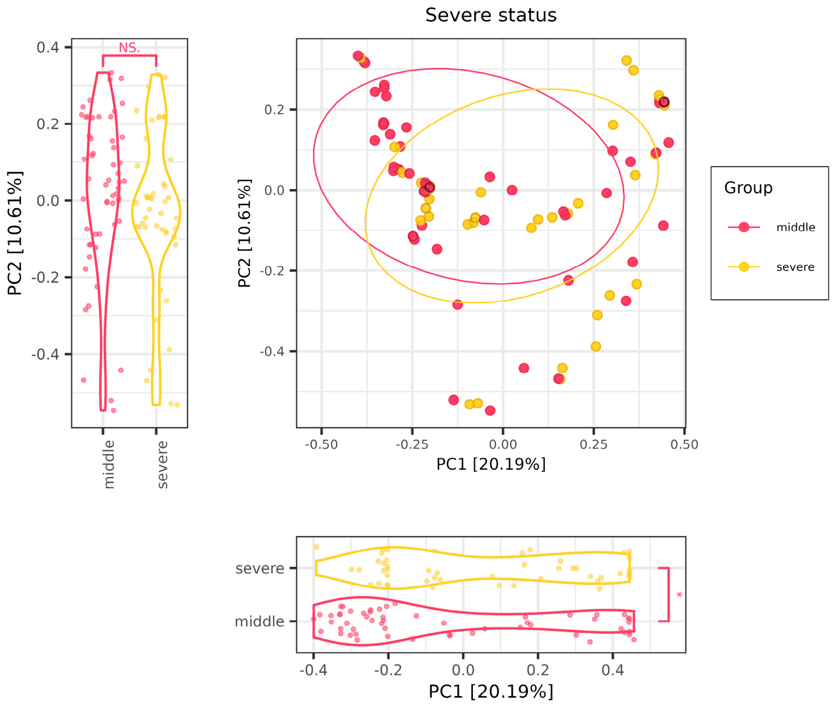


Figure S10 PCoA with Bray–Curtis distance decomposition for samples from different severity groups (NS. - p-value > 0.05, * - p-value ≤ 0.05).

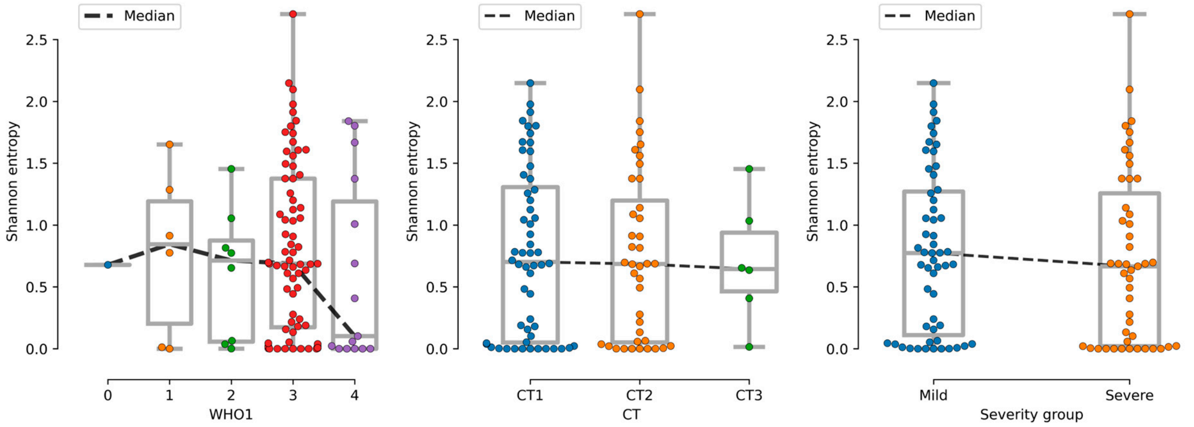


Figure S11 Changes in the trend of alpha diversity depending on the severity of the disease (ASV level). CT is computed tomography score (CT1 - less than 25-50% of the lungs are affected; CT2 - moderate pneumonia, 25-50% of the lungs are affected; CT3 - 50-75% of the lungs are affected; CT4 is a severe form of pneumonia, affecting >75%). who1 score is WHO performance scores at admission to the hospital. Severity group is a combined score of disease severity according to CT and the WHO scale.

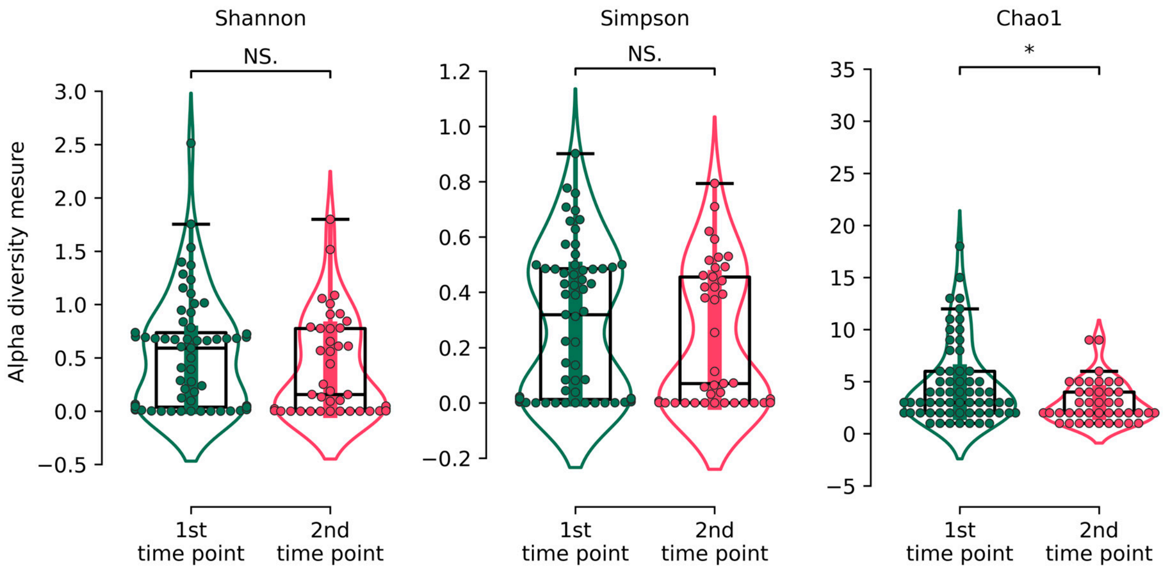


Figure S12 Alpha diversity for different time points for Species Level (Mann-Whitney U-test results : ** - p-value ≤ 0.01, *** - p-value ≤ 0.001).
