# Supplementary material for: Gut Mycobiome Changes During COVID-19 Disease
Source: J Fungi (Basel). 2025 Mar 3;11(3):194. doi: 10.3390/jof11030194 (PMC11943151; doi:10.3390/jof11030194)
Supplement: Supplementary file 1 [file jof-11-00194-s001.zip › Supplementary2.pdf]

Supplementary 2

>ASV0
TTTCCGTAGGTGAACCTGCGGAAGGATCATTACTGATTTGCTTAATTGCACCACATGTGTTTTTCTTTGA
AACAAACTTGCTTTGGCGGTGGGCCCAGCCTGCCGCCAGAGGTCTAAACTTACAACCAATTTTTTATCAA
CTTGTCACACNNNNNNNNNNTGGGTTTGCTTGAAAGACGGTAGTGGTAAGGCGGGATCGCTTTGACAA
TGGCTTAGGTCTAACCAAAAACATTGCTTGCGGCGGTAACGTCTACCACGTATATCTTCAAACTTTGACC
TCAAATCAGGTAGGACTACCCGCTGAACTTAA
>ASV1
TTTCCGTAGGTGAACCTGCGGAAGGATCATTACTGATTTGCTTAATTGCACCACATGTGTTTTTCTTTGA
AACAAACTTGCTTTGGCGGTGGGCCCAGCCTGCCGCCAGAGGTCTAAACTTACAACCAATTTTTTATCAA
CTTGTCACACNNNNNNNNNNTGGGTTTGCTTGAAAGACGGTAGTGGTAAGGCGGGATCGCTTTGACAA
TGGCTTAGGTCTAACCAAAAACATTGCTTGCGGCGGTAGCGTCTACCACGTATATCTTCAAACTTTGACC
TCAAATCAGGTAGGACTACCCGCTGAACTTAA
>ASV3
TTTCCGTAGGTGAACCTGCGGAAGGATCATTACTGATTTGCTTAATTGCACCACATGTGTTTTTCTTTGA
AACAAACTTGCTTTGGCGGTGGGCCCAGCCTGCCGCCAGAGGTCTAAACTTACAACCAATTTTTTATCAA
CTTGTCACACNNNNNNNNNNTGGGTTTGCTTGAAAGACGGTAGTGGTAAGGCGGGATCGCTTTGACAA
TGGCTTAGGTCTAACCAAAAACATTGCTTGCGGCGGTAACGTCCACCACGTATATCTTCAAACTTTGACC
TCAAATCAGGTAGGACTACCCGCTGAACTTAA
>ASV4
TTTCCGTAGGTGAACCTGCGGAAGGATCATTACCTAGAGTTTGTAGACTTCGGTCTGCTACCTCTTACCC
ATGTCTTTTGAGTACCTTCGTTTCCTCGGCGGGTCCGCCCGCCGATTGGACAACATTCAAACCCTTTGCA
GTTGCAATCANNNNNNNNNNGCGTGTAGACTCGCCTTAAAACAATTGGCAGCCGGCGTATTGATTTCG
GAGCGCAGTACATCTCGCGCTTTGCACTCACAACGACGACGTCCAAAAGTACATTTTTACACTCTTGACC
TCGGATCAGGTAGGGATACCCGCTGAACTTAA
>ASV5
TTTCCGTAGGTGAACCTGCGGAAGGATCATTACTGATTTGCTTAATTGCACCACATGTGTTTTTCTTTGA
AACAAACTTGCTTTGGCGGTGGGCCCAGCCTGCCGCCAGAGGTCTAAACTTACAACCAATTTTTTATCAA
CTTGTCACACNNNNNNNNNNTGGGTTTGCTTGAAAGACGGTAGTGGTAAGGCGGGATCGCTTTGACAA
TGGCTTAGGTCTAACCAAAAACATTGCTTGCGGCGGCAACGTCTACCACGTATATCTTCAAACTTTGACC
TCAAATCAGGTAGGACTACCCGCTGAACTTAA
>ASV6
TTTCCGTAGGTGAACCTGCGGAAGGATCATTACAGAATGAAAAGTGCTTAACTGCATTTTTTCTTACACA
TGTGTTTTTCTTTTTTTGAAAACTTTGCTTTGGTAGGCCTTCTATATGGGGCCTGCCAGAGATTAAACTCA
ACCAAATTTNNNNNNNNNNTCGGGTTTGGTGTTGAGCGATACGCTGGGTTTGCTTGAAAGAAAGGCGG
AGTATAAACTAATGGATAGGTTTTTTCCACTCATTGGTACAAACTCCAAAACTTCTTCCAAATTCGACCT
CAAATCAGGTAGGACTACCCGCTGAACTTAA
>ASV7
TTTCCGTAGGTGAACCTGCGGAAGGATCATTACTGATTTGCTTAATTGCACCACATGTGTTTTTCTTTGA
AACAAACTTGCTTTGGCGGTGGGCCCAGCCTGCCGCCAGAGGTCTAAACTTACAACCAATTTTTTATTAA
CTTGTCACACNNNNNNNNNNTGGGTTTGCTTGAAAGACGGTAGTGGTAAGGCGGGATCGCTTTGACAA
TGGCTTAGGTCTAACCAAAAACATTGCTTGCGGCGGTAACGTCCACCACGTATATCTTCAAACTTTGACC
TCAAATCAGGTAGGACTACCCGCTGAACTTAA
>ASV8
TTTCCGTAGGTGAACCTGCGGAAGGATCATTATGAATTATAAATATTTGTGAATTTACCACAGCAAACAA
AAATCATACAATCAATAATTAAATAATTAAAACTTTTAACAATGGATCTCTTGGTTCTCGTATCGATGAA
GAACGCAGCGNNNNNNNNNNATTGCACTTTGGGGTATCCCCCAAAGTATACTTGTTTGAGCGTTGTTTC
TCTCTTGGAATTGCTTTGCTCTTCTAAAATTTCGAATCAAATTCGTTTGAAAAACAACACTATTCAACCTC
AGATCAAGTAGGATTACCCGCTGAACTTAA

>ASV9
TTTCCGTAGGTGAACCTGCGGAAGGATCATTATGAATTAATAATATTTGTGAAATTTCAACAAACAACAA
CAATCATACAATCAATAATTAAAAAATTAAAACTTTTAACAATGGATCTCTTGGTTCTCGTATCGATGAA
GAACGCAGCGNNNNNNNNNNNATTGCACTTTGGGGTATCCCCCAAAGTATACTTGTTTGAGCGTTGTTTC
TCTCTTGGAATTGCTTTGCTCTTCTAAAATTTCGAATCAAATTCGTTTGAAAAACAACACTATTCAACCTC
AGATCAAGTAGGATTACCCGCTGAACTTAA
>ASV10
TCTCCGTTGGTGAACCAGCGGAGGGATCATTACCGAGTTTACAACTCCCAAACCCATGTGAACATACCTT
ACAGTTGCTTCGGCGGAGCCGCCCCGGCGCCCGGAACCCAGTTTCGCGGCCCGGACCAAGGCGCCCGC
CGGAGGCCACAANNNNNNNNNNNCCGCCGGCCCCGAAATGAAGTGGCGGCCCGTCCGCGGCGACCTCT
GCGTAGTAACTCCACTCGCACCGGGACCCGGGCGCGGCCACGCCGTAAAACCCCCAACTTCCGAATGTT
GACCTCGAATCAGGTAGGAATACCCGCTGAACTTAA
>ASV11
TTTCCGTAGGTGAACCTGCGGAAGGATCATTACTGATTTGCTTAATTGCACCACATGTGTTTTTCTTTGA
AACAAACTTGCTTTGGCGGTGGGCCCAGCCTGCCGCCAGAGGTCTAAACTTACAACCAATTTTTTATCAA
CTTGTCACACNNNNNNNNNNNTGGGTTTGCTTGAAAGACGGTAGTGGTAAGGCGGGATCGCTTTGACAA
TGGCTTAGGTCTAACCAAAAACATTGTTTGCGGCGGTAACGTCCACCACGTATATCTTCAAACTTTGACC
TCAAATCAGGTAGGACTACCCGCTGAACTTAA
>ASV12
TTTCCGTAGGTGAACCTGCGGAAGGATCATTATGAATTAATAATATTTGTGAAATTTCAACAAACAACAA
TAATCATACAATCAATAATTAAAAAATTAAAACTTTTAACAATGGATCTCTTGGTTCTCGTATCGATGAA
GAACGCAGCGNNNNNNNNNNNATTGCACTTTGGGGTATCCCCCAAAGTATACTTGTTTGAGCGTTGTTTC
TCTCTTGGAATTGCATTGCTCTTCTAAAATTTCGAATCAAATTCGTTTGAAAAACAACACTATTCAACCTC
AGATCAAGTAGGATTACCCGCTGAACTTAA
>ASV13
TTTCCGTAGGTGAACCTGCGGAAGGATCATTATGAATTATAAATATTTGTGAATTTACCACAGCAAACAT
CAATCATACAATCAATAATTAAAAAATTAAAACTTTTAACAATGGATCTCTTGGTTCTCGTATCGATGAA
GAACGCAGCGNNNNNNNNNNNATTGCACTTTGGGGTATCCCCCAAAGTATACTTGTTTGAGCGTTGTTTC
TCTCTTGGAATTGCTTTGCTCTTCTAAAATTTCGAATCAAATTCGTTTGAAAAACAACACTATTCAACCTC
AGATCAAGTAGGATTACCCGCTGAACTTAA
>ASV14
TTTCCGTAGGTGAACCTGCGGAAGGATCATTACTGATTTGCTTAATTGCACCACATGTGTTTTTCTTTGA
AACAAACTTGCTTTGGCGGTGGGCCCAGCCTGCCGCCAGAGGTCTAAACTTACAACCAATTTTTTATTAA
CTTGTCACACNNNNNNNNNNNTGGGTTTGCTTGAAAGACGGTAGTGGTAAGGCGGGATCGCTTTGACAA
TGGCTTAGGTCTAACCAAAAACATTGCTTGCGGCGGTAACGTCTACCACGTATATCTTCAAACTTTGACC
TCAAATCAGGTAGGACTACCCGCTGAACTTAA
>ASV15
TTTCCGTAGGTGAACCTGCGGAAGGATCATTAAGAATTAATAATATTTGTGAAATTTCAACAAACAACAA
TAATCATACAATCAATAATTAAAAAATTAAAACTTTTAACAATGGATCTCTTGGTTCTCGTATCGATGAA
GAACGCAGCGNNNNNNNNNNNATTGCACTTTGGGGTATCCCCCAAAGTATACTTGTTTGAGCGTTGTTTC
TCTCTTGGAATTGCTTTGCTCTTCTAAAATTTCGAATCAAATTCGTTTGAAAAACAACACTATTCAACCTC
AGATCAAGTAGGATTACCCGCTGAACTTAA
>ASV16
TCTCCGTAGGTGAACCTGCGGAGGGATCATTACAAGAACGCCCGGGCTTCGGCCTGGTTATTCATAACC
CTTTGTTGTCCGACTCTGTTGCCTCCGGGGCGACCCTGCCTTCGGGCGGGGGCTCCGGGTGGACACTTC
AAACTCTTGCGTNNNNNNNNNNNCGCCGCGTGCCTCAAATCGTCCGGCTGGGTCTTCTGTCCCCTAAGC
GTTGTGGAAACTATTCGCTAAAGGGTGTTCGGGAGGCTACGCCGTAAAACAACCCCATTTCTAAGGTTG
ACCTCGGATCAGGTAGGGATACCCGCTGAACTTAA
>ASV18

TTTCCGTAGGTGAACCTGCGGAAGGATCATTAAAGAGTAAGGGTGCTCAGCGCCCGACCTCCAACCCTT
TGTTGTTAAAACTACCTTGTTGCTTTGGCGGGACCGCTCGGTTCCGAGCCGCTGGGGATTCGTCCCAGG
CGAGTGCCCGCCNNNNNNNNNNGCGCGCCTTAAAGACCTCGGCGAGGCCTCCCCGGCTTTAGGCGTAG
TAGAATTTATTCGAACGTCTGTCAAAGGAGAGGAACTCTGCCGACTGAAACCTTTATTTTTACAGGTTGA
CCTCGGATCAGGTAGGGATACCCGCTGAACTTAA
>ASV19
TCTCCGTAGGTGAACCTGCGGAGGGATCATTACAAGTGACCCCGGTCTAACCACCGGGATGTTCATAAC
CCTTTGTTGTCCGACTCTGTTGCCTCCGGGGCGACCCTGCCTTCGGGCGGGGGCTCCGGGTGGACACTT
CAAACTCTTGCGNNNNNNNNNNCGCCGCGTGCCTCAAATCGACCGGCTGGGTCTTCTGTCCCCTAAGC
GTTGTGGAAACTATTCGCTAAAGGGTGTTCGGGAGGCTACGCCGTAAAACAACCCCATTTCTAAGGTTG
ACCTCGGATCAGGTAGGGATACCCGCTGAACTTAA
>ASV20
TTTCCGTAGGTGAACCTGCGGAAGGATCATTATCGATACTATACTTTACTGTGGAACTTTATTCGTTTAA
CTAATCATCCAACTAACTAATAACTATCAACAACGGATCTCTTGGCTCTCACATCGATGAAGAACGCAGC
GAACCGCGATNNNNNNNNNNCTTTGAACGCACATTGCGCGGTGTGGTATTCCGCACCGCACGGATGGA
AGAGCGTGTTCCCTTTGGGATCGCATTGCTTTCTTGAAATGAATCTATTACTTCAATAAACTCATTTCAC
CTCTTTCATCCGAGATTACCCGCTGAACTTAA
>ASV21
TTTCCGTAGGTGAACCTGCGGAAGGATCATTACCGAGTGCGGGCCCTCTGGGTCCAACCTCCCATCCGT
GTCTATCTGTACCCTGTTGCTTCGGCGTGGCCACGGCCCGCCGGAGACTAACATTTGAACGCTGTCTGA
AGTTTGCAGTCTNNNNNNNNNNGCCCAAAAGGCAGTGGCGGCACCATGTCTGGTCCTCGAGCGTATGG
GGCTTTGTCACCCGCTCCCGTAGGTCCAGCTGGCAGCTAGCCTCGCAACCAATCTTTTTAACCAGGTTGA
CCTCGGATCAGGTAGGGATACCCGCTGAACTTAA
>ASV22
TTTCCGTAGGTGAACCTGCGGAAGGATCATTACCGAGTGAGGGCCCTCTGGGTCCAACCTCCCACCCGT
GTTTATTTTACCTTGTTGCTTCGGCGGGCCCGCCTTAACTGGCCGCCGGGGGGCTTACGCCCCCGGGCC
CGCGCCCGCCGANNNNNNNNNNGGGCCCGAAAGGCAGCGGCGGCACCGCGTCCGGTCCTCGAGCGTA
TGGGGCTTTGTCACCCGCTCTGTAGGCCCGGCCGGCGCTTGCCGATCAACCCAAATTTTTATCCAGGTTG
ACCTCGGATCAGGTAGGGATACCCGCTGAACTTAA
>ASV23
TTTCCGTAGGTGAACCTGCGGAAGGATCATTAAATAATCAATAATTTTGGCTTGTCCATTATTATCTATTT
ACTGTGAACTGTATTATTACTTGACGCTTGAGGGATGCTCCACTGCTATAAGGATAGGCGGTGGGGATGT
TAACCGAGTNNNNNNNNNNTGAAATGTACAAAGGCCTGATCTTGTTTAAATGCCTGAACTTTTTTTTAA
TATAAAGAGAAGCTCTTGCGGTAAACTGTGCTGGGGCCTCCCAAATAATACTCTTTTTAAATTTGATCTG
AAATCAGGCGGGATTACCCGCTGAACTTAA
>ASV24
TTTCCGTAGGTGAACCTGCGGAAGGATCATTAAAATAATACTTACACTTTGCATTTGCGAACAAAAAAAT
AAATCTTTTATTCGAATTTCTTAATATCAAAACTTTCAACAACGGATCTCTTGGTTCTCGCATCGATGAAG
AACGCAGCGNNNNNNNNNNCGAGGCATTCCTCGAGGCATGCCTGTTTGAGCGTCGCATCCCCTCTAAC
CCCCGGTTAGGCGTTGCTCCGAAATATCAACCGCGCTGTCAAACACGTTTACAGCACGACATTTCGCCCT
CAAATCAGGTAGGACTACCCGCTGAACTTAA
>ASV25
TTTCCGTAGGTGAACCTGCGGAAGGATCATTAAGAATTGAAAATATTTGTGAAATTACCACAGCAAACAA
TAATCATACAATCAAAACAAAAATAATCAAAACTTTTAACAATGGATCTCTTGGTTCTCGTATCGATGAA
GAACGCAGCGNNNNNNNNNNATTGCACTTTGGGGTATCCCCCAAAGTATACTTGTTTGAGCGTTGTTTC
TCTCTTGGAATTGCATTGCTTTTCTAAAAAATCGAATCAAATTCGTTTGAAAAACAACACTATTCAACCT
CAGATCAAGTAGGATTACCCGCTGAACTTAA
>ASV26
TCTCCGTAGGTGAACCTGCGGAGGGATCATTACACAAATATGAAGGCGGGCTGGAACCTCTCGGGGTTA
CAGCCTTGCTGAATTATTCACCCTTGTCTTTTGCGTACTTCTTGTTTCCTTGGTGGGTTCGCCCACCACTA

GGACAAACATNNNNNNNNNNTGGAGACTCGCCTTAAAGTAATTGGCAGCCGGCCTACTGGTTTCGGAG
CGCAGCACAAGTCGCACTCTCTATCAGCAAAGGTCTAGCATCCATTAAGCCTTTTTTTCAACTTTTGACC
TCGGATCAGGTAGGGATACCCGCTGAACTTAA
>ASV27
TTTCCGTAGGTGAACCTGCGGAAGGATCATTAAAATAATACTTACACTTTGCATTTGCGAACAAAAAAAA
GAACATTACACTTCTAATATATTTTTATCAAAACTTTCAACAACGGATCTCTTGGTTCTCGCATCGATGAA
GAACGCAGCNNNNNNNNNNCGAGGCATTCCTCGAGGCATGCCTGTTTGAGCGTCGCATCCCCTCTAAC
CCCCGGTTAGGCGTTGCTCCGAAATATCAACCGCGCTGTCAAACACGTTTACAGCACGACATTTCGCCCT
CAAATCAGGTAGGACTACCCGCTGAACTTAA
>ASV28
TCTCCGTAGGTGAACCTGCGGAGGGATCATTACACAATATGAAAGCGGGTTGGGACCTCACCTCGGTGA
GGGCTCCAGCTTGTCTGAATTATTCACCCATGTCTTTTGCGCACTTCTTGTTTCCTGGGCGGGTTCGCCC
GCCACCAGGACNNNNNNNNNNAGACTCGCCTTAAAATGATTGGCAGCCGACCTACTGGTTTCGGAGCG
CAGCACAATTCTTGCACTTTGAATCAGCCTTGGTTGAGCATCCATCAAGACCACATTTTTAACTTTTGAC
CTCGGATCAGGTAGGGATACCCGCTGAACTTAA
>ASV29
TTTCCGTAGGTGAACCTGCGGAAGGATCATTACTGATTTGCTTAATTGCACCACATGTGTTTTTCTTTGA
AACAAACTTGCTTTGGCGGTGGGCCCAGCCTGCCGCCAGAGGTCTAAACTTACAACCAATTTTTTATTAA
CTTGTCACACNNNNNNNNNNTGGGTTTGCTTGAAAGACGGTAGTGGTAAGGCGGGATCGCTTTGACAA
TGGCTTAGGTCTAACCAAAAACATTGTTTGCGGCGGTAACGTCCACCACGTATATCTTCAAACTTTGACC
TCAAATCAGGTAGGACTACCCGCTGAACTTAA
>ASV30
TTTCCGTAGGTGAACCTGCGGAAGGATCATTACTAAATGCGAAGCTTCGGCTTAGCTTTAATAATCCCTC
AACACCTGTGCACCGTATTGTCTTCGGACAATTACAATTACAAACATCAGTGTAAAGAATGTATTATTAC
TTTAACAAAANNNNNNNNNNGTTTCCGATCTAGGTGGTTTGGACGTGTGCCATATTATTTGGCTCGTCT
TAAATGTCTAAGTGGAATGCGTAATAAGTTTTTCGTAACCTCCACTCTAATACACTTTAAGCTCTGACCT
CAAATCAGGTAGGACTACCCGCTGAACTTAA
>ASV31
TCTCCGTTGGTGAACCAGCGGAGGGATCATTGCTGGAACAAACGCCCTCACGGGTGCTACCCAGAAACC
CTTTGTGAACTATACTTAATCGTTGCCTCGGCATTGGTTGACTTTGAATAAAGTCCCTGTAGGCCCTTCT
AGGGCTTTCAGNNNNNNNNNNTACCCTCTGAAATTTAGTGGCGGGCTCGCTAGAATTTTGAGCGTAGTA
ATTTTACCTCGTTTTTAAAGACTAGTGGGACTTCTTGCCGTAAAACCCCCCAACTTTCTGAAATTTGACC
TCGGATCAGGTAGGAATACCCGCTGAACTTAA
>ASV32
TCTCCGTAGGTGAACCTGCGGAGGGATCATTACACAATAACAAGGCGGGCTGGACACCCCCCGCTGGGC
ACTGCTTCACGGCGTGCGCGGCGGGGCCGGCCCTGCTGAATTATTCACCCGTGTCTTTTGCGTACTTCTT
GTTTCCTGGGTNNNNNNNNNNCTGGAGACTCGCCTTAAAGTCATTGGCAGCCGGCCTACTGGTTTCGG
AGCGCAGCACAAGTCGCGCTCTTTGCCAGCCAAGGTCAGCGTCCAGCAAGCCTTTTTTTCAACCTTTGAC
CTCGGATCAGGTAGGGATACCCGCTGAACTTAA
>ASV35
TTTCCGTAGGTGAACCTGCGGAAGGATCATTAAAATTAATTTATTACATTGTTTTTGAAGCAAACACTAA
TAACCATTAATATTCAATAACTTAGAAAAATCAAAACTTTCAACAACGGATCTCTTGGTTCTCGCATCGA
TGAAGAACGCNNNNNNNNNNGTATTCCTCAAGGCATGCCTGTTTGAGCGTCGGCTCCCTTCAAACCCA
CGGGTTTGGTGTTGCCTTCCGAAATATCACAGTTGTTGCAATACGTTACTTCAACTTTATTCTTTCGCCCT
CAAATCAGGTAGGACTACCCGCTGAACTTAA
>ASV36
TTTCCGTAGGTGAACCTGCGGAAGGATCACTAGTGATTAAATCGAGCGTGTCTTCATTGACCGCTCACCC
TTCTCACCATCCACATACACCTGTGCACTGTTTAGCCTGAGCCGGTTTTCCGGTCCAGGTTATCATTTCA
TACAAACTCTNNNNNNNNNNTCCACTCAACGTGATAAGTATTTCGTTGAGGACAGTTGCAGCAATGCGG

CTGGCCGGGATAAGAAAGGCATAGTTGTCAGCTTCTAATCGCCCTTGGGCAATTTTTTATGATCTGGCCT
CAAATCAGGTAGGACTACCCGCTGAACTTAA
>ASV38
TTTCCGTAGGTGAACCTGCGGAAGGATCATTAAAATTAATTTATTACATTGTTTTTGAAGCAAACACTAA
TAACCATTAATATTCAATAACTTAGAAAAATCAAAACTTTCAACAACGGATCTCTTGGTTCTCGCATCGA
TGAAGAACGCNNNNNNNNNNGTATTCCTCAAGGCATGCCTGTTTGAGCGTCGGCTCCCTTCAAACCCC
CGGGTTTGGTGTTGCCTTCCGAAATATCACAGTTGTTGCAATACGTTACTTCAACTTTATTCTTTCGCCCT
CAAATCAGGTAGGACTACCCGCTGAACTTAA
>ASV39
TTTCTGTAGGTGAACCTGCAGAAGGATCATTAGTGAAGATTCAAGGGCCAGCCATACAGACGTACAATA
AGTGTGTCTCTGGCGGCTCGCATCCACTATACATCCATAAACCCGTGTGCACTGTTCTAAGGAGTAAGAA
AGAAGAAGAGANNNNNNNNNNCATAGCATGATACGTCATTTGCTATGCTGTAGGAGAGCATTTGGTTG
TGGTTATACCGCGTGCGTCATTTTTTTTTCTTGCAAAGAGGAAAAAAAAAGCCCCCCTTTCATTTCTGGT
CTCAAATCAGGTAGGATCACCCGCTGAACTTAA
>ASV40
TTTCCGTAGGTGAACCTGCGGAAGGATCATTAGTGAATTGCTCTTTGAGCGTTAACTATATCCATCTACA
CCTGTGAACTGTTGATTGATTTCGGTCAATTACTTTACAAACATTGTGTAATGAACGTCATGTTATTATAA
CAAAAATAANNNNNNNNNNNTGGCTCACCTTAAAAGAGTTAGCGTGTTTAACTTGTCGATCTGGCGTAAT
AAGTTTCGCTGGTGTAGACTTGAGAAGTGCGCTTCTAATCGTCCTCGGACAATTCTTGAACTCTGGTCTC
AAATCAGGTAGGACTACCCGCTGAACTTAA
>ASV41
TCTCCGTTGGTGAACCAGCGGAGGGATCATTACCGAGTTTACAACTCCCAAACCCCTGTGAACATACCA
TATGTTGCCTCGGCGGATCAGCCCGTCCTTCGGGACGGCCCGCCGCAGGACCCTAAACTCTGTTTTTAG
TGGAACTTCTGANNNNNNNNNNNAGAACACTCCCCAAATTGATTGGCGGTCACGTCGAGCTTCCATAGC
GTAGTAATTTACACATCGTTACTGGTAATCGTCGCGGCCACGCCGTTAAACCCCAACTTCTGAATGTTGA
CCTCGGATCAGGTAGGAATACCCGCTGAACTTAA
>ASV42
TTTCCGTAGGTGAACCTGCGGAAGGATCATTATGAATTAATAATATTTGTGAATTTACCACAACAAACAT
CAATCATACAATCAATAATTAAAAAATTAAAACTTTTAACAATGGATCTCTTGGTTCTCGTATCGATGAA
GAACGCAGCGNNNNNNNNNNATTGCACTTTGGGGTATCCCCCAAAGTATACTTGTTTGAGCGTTGTTTC
TCTCTTGGAATTGCTTTGCTCTTCTAAAATTTCGAATCAAATTCGTTTGAAAAACAACACTATTCAACCTC
AGATCAAGTAGGATTACCCGCTGAACTTAA
>ASV43
TTTCCGTAGGTGAACCTGCGGAAGGATCATTACCGAGTGTAGGGTTCCTAGCGAGCCCAACCTCCCACC
CGTGTTTACTGTACCTTAGTTGCTTCGGCGGGCCCGCCATTCATGGCCGCCGGGGGCTCTCAGCCCCGG
GCCCGCGCCCGCNNNNNNNNNNGGCCCCAAAGGCAGCGGCGGCACCGCGTCCGATCCTCGAGCGTAT
GGGGCTTTGTCACCCGCTCTGTAGGCCCGGCCGGCGCTTGCCGAACGCAAATCAATCTTTTTCCAGGTT
GACCTCGGATCAGGTAGGGATACCCGCTGAACTTAA
>ASV44
TTTCCGTAGGTGAACCTGCGGAAGGATCATTAAAGAGTAAGGGTGCTCAGCGCCCGACCTCCAACCCTT
TGTTGTTAAAACTACCTTGTTGCTTTGGCGGGACCGCTCGGTTCCGAGCCGCTGGGGATTCGTCCCAGG
CGAGTGCCCGCCNNNNNNNNNNCGCGCCTTAAAGACCTCGGCGAGGCCTCACCGGCTTTAGGCGTAGT
AGAATTTATTCGAACGTCTGTCAATGGAGAGGACTTCTGCCGACTGAAACCTTTATTTTTTACAGGTTGA
CCTCGGATCAGGTAGGGATACCCGCTGAACTTAA
>ASV45
TTTCCGTAGGTGAACCTGCGGAAGGATCATTATCGAGTTTTGAAATGGGTTGTAGCTGGCTTCTCCGGAG
GCATGTGCACGCCCTGCTCATCCACTCTACACCTGTGCACTTACTGTGGGTATCGGGAGGTGTCGCGTC
GTTTACGGCGANNNNNNNNNNNTTAGCTTGATTCCTTGCGGATCGGCTCTCGGTGTGATAATTGTCTACG
CCGTGACCGTGAAGCGTTTTGGCAAGCTTCTAACCGTCTCTAACGAGACAGCTTACTTTGACCTCTGACC
TCAAATCAGGTAGGACTACCCGCTGAACTTAA

>ASV48
TTTCCGTAGGTGAACCTGCGGAAGGATCATTACCGAGTGAGGGCCCTCTGGGTCCAACCTCCCACCCGT
GTTTATTTTACCTTGTTGCTTCGGCGAGCCTGCCTTCGGGCTGCCGGGGGGCATCTGCCCCCGGGTCCG
CGCTCGCCGGAGNNNNNNNNNNCGGGCCCGAAAGGCAGCGGCGGCACCGCGTCCGGTCCTCGAGCGT
ATGGGGCTTTGTCACCCGCTCTGTAGGACTGGCCGGCGCCTGCCGATCAACCAAACTTTTTTCCAGGTTG
ACCTCGGATCAGGTAGGGATACCCGCTGAACTTAA
>ASV49
TTTCCGTAGGTGAACCTGCGGAAGGATCATTACAGTATTCTTTTTGCCAGCGCTTAATTGCGCGGCGAAA
AAACCTTACACACAGTGTTTTTTGTTATTACAAGAACTTTTGCTTTGGTCTGGACTAGAAATAGTTTGGG
CCAGAGGTTTNNNNNNNNNNACTGGATAGTGCTATATGACTTTCAATGTATTAGGTTTATCCAACTCGT
TGAATAGTTTAATGGTATATTTCTCGGTATTCTAGGCTCGGCCTTACAATATAACAAACAAGTTTGACCT
CAAATCAGGTAGGATTACCCGCTGAACTTAA
>ASV50
TTTCCGTAGGTGAACCTGCGGAAGGATCATTATCGAGTTTTGAACGGGTTGTCTGCTGGCTCGCAAGGG
CATGTGCACGCCTGTCTCATCCACTCTCAACTTCTGTGCACTTTTCATAGGCCGGCTTGTGGGTGCGTTC
GCGCACTTGTANNNNNNNNNNAATGCATTAGTGCGAATGTTACCAGCCGCTTCAGCGTGATAATTATCT
GCGTTGCTGTGGAGGGTATTCTAGTGTTCGCGCTTCTAACCGTCTTCGGACAAATTTCTGAACTCTGAGC
TCAAATCAGGTAGGACTACCCGCTGAACTTAA
>ASV51
TCTCCGTAGGTGAACCTGCGGAGGGATCATTACACAACAAAATATGAAGGCCTGGCTTCGCGGCCGGCT
GAAATATTTTTTTCACCCATGTCTTTTGCGCACTTGTTGTTTCCTGGGCGGGTTCGCCCGCCACCAGGAC
CAAACCATAAANNNNNNNNNNCGCCTTAAAACGATTGGCAGCCGGCCTACTGGTTTCGGAGCGCAGCA
CATATTTTGCGCTTTGTATCAGGAGAAAAGGACGGTAATCCATCAAGACTCTACATTTTTAACTTTTGAC
CTCGGATCAGGTAGGGATACCCGCTGAACTTAA
>ASV52
TTTCCGTAGGTGAACCTGCGGAAGGATCATTACTGAGTGAGGGCCCTCTGGGTCCAACCTCCCACCCGT
GTTTATTTACCTTGTTGCTTCGGCGGGCCCGCCTTAACTGGCCGCCGGGGGGCTTACGCCCCCGGGCCC
GCGCCCGCCGAANNNNNNNNNNGGGCCCGAAAGGCAGCGGCGGCACCGCGTCCGGTCCTCGAGCGTA
TGGGGCTTTGTCACCCGCTCTGTAGGCCCGGCCGGCGCTTGCCGATCAACCCAAATTTTTATCCAGGTTG
ACCTCGGATCAGGTAGGGATACCCGCTGAACTTAA
>ASV53
TTTCCGTAGGTGAACCTGCGGAAGGATCATTAGTGATTGCCTTAATTGGCTTATAACTATATCCACTTAC
ACCTGTGAACTGTTCTACTACTTGACGCAAGTCGAGTATTTTTACAAACAATGTGTAATGAACGTCGTTT
TATTATAACANNNNNNNNNNTCGCCTTAAAAGAGTTAGCAAGTTTGACATTAATGTCTGGTGTAATAAG
TTTCACTGGGTCCATTGTGTTGAAGCGTGCTTCTAATCGTCCGCAAGGACAATTACTTTGACTCTGGCCT
GAAATCAGGTAGGACTACCCGCTGAACTTAA
>ASV56
TTTCCGTAGGTGAACCTGCGGAAGGATCACTAGTGATTAAATCGAGCGTGTCTTCATTGACCGCTCACCC
TTCTCACCATCCACATACACCTGTGCACTGTTTAGCCTGAGCCGGTTTTCCGGTCCAGGTTATCATTTCA
TACAAACTCTNNNNNNNNNNCCACTCAACGTGATAAGTATTTCGTTGAGGACAGTTGCAGCAATGCGGC
TGGCCGGGATAAGAAAGGCATAGTTGTCAGCTTCTAATCGCCCTTGGGCAATTTTTTTATGATCTGGCCT
CAAATCAGGTAGGACTACCCGCTGAACTTAA
>ASV57
TTTCCGTAGGTGAACCTGCGGAAGGATCATTACCGAGTGCGGGCCCCTCGGGGCCCAACCTCCCACCCG
TGTTGCCCGAACCTATGTTGCCTCGGCGGGCCCCGCGCCCGCCGACGGCCCCCCTGAACGCTGTCTGAA
GTTGCAGTCTGANNNNNNNNNNCCCGAAAGGCAGCGGCGGCACCGCGTCCGGTCCTCGAGCGTATGGG
GCTTCGTCACCCGCTCTAGTAGGCCCGGCCGGCGCCAGCCGACCCCCAACCTTTAATTATCTCAGGTTG
ACCTCGGATCAGGTAGGGATACCCGCTGAACTTAA
>ASV60

TTTCCGTAGGTGAACCTGCGGAAGGATCATTAAAAAGAATTATACACTTTGCATTTGCGAACAAAAAAAT
AAATTTTTTTATTCGAATCATTTAAATCAAAACTTTCAACAACGGATCTCTTGGTTCTCGCATCGATGAAG
AACGCAGCGNNNNNNNNNNCGAGGCATTCCTCGAGGCATGCCTGTTTGAGCGTCGCATCCCCTCTAAC
CCCCGGTTAGGCGTTGCTCCGAAATATCAACCGCGCTGTCAAACACGTTTACAGCACGACATTTCGCCCT
CAAATCAGGTAGGACTACCCGCTGAACTTAA
>ASV61
TTTCCGTAGGTGAACCTGCGGAAGGATCATTACAGTATTCTTTTGCCAGCGCTTAACTGCGCGGCGAAAA
ACCTTACACACAGTGTCTTTTTGATACAGAACTCTTGCTTTGGTTTGGCCTAGAGATAGGTTGGGCCAGA
GGTTTAACAANNNNNNNNNNGTACTAGATAGTGCTGTCGACCTCTCAATGTATTAGGTTTATCCAACTC
GTTGAATGGTGTGGCGGGATATTTCTGGTATTGTTGGCCCGGCCTTACAACAACCAAACAAGTTTGACCT
CAAATCAGGTAGGAATACCCGCTGAACTTAA
>ASV62
TTTCCGTAGGTGAACCTGCGGAAGGATCATTAAGAATTGATAATTAATTGTGAAAATTATACAGCAAACA
ATAATTTTATAGTCAAAACAAAAAAAAATTAAAACTTTTAACAATGGATCTCTTGGTTCTCGTATCGATG
AAGAACGCAGNNNNNNNNNNATTGCACTTTGGGGTATCCCCCAAAGTATACTTGTTTGAGCGTTGTTTC
TCTCTTGGAATTGCATTGCTTTTCTAAAATATTGAATCAAATTCGTTTGAAAAACAACACTATTCAACCTC
AGATCAAGTAGGATTACCCGCTGAACTTAA
>ASV63
TTTCTGTAGGTGAACCTGCAGAAGGATCATTAGTGAAGATTTGGGCAGGCCATACGGACGCCAAAAAGT
GTCCCTGGCCGCCTACACCCACTATACATCCACAAACCCGTGTGCACTGTCTTGGAGAAAGGCTTCAGA
GAAGTTTTTTGTNNNNNNNNNNTTTCTCCTGGCATGGCATGATACGTCATTTGCTATGTCGCCTAAAGG
AGGAATGTTTGGTTGTGTCTGCGTGTGCTTCGAACTTGCCTCTGTGGCACATCCCAATTTCACTTCTGGT
CTCAAATCAGGTAGGATCACCCGCTGAACTTAA
>ASV64
TTTCCGTAGGTGAACCTGCGGAAGGATCATTACCGAGTGCGGGTCCTTTGGGCCCAACCTCCCATCCGT
GTCTATTATACCCTGTTGCTTCGGCGGGCCCGCCGCTTGTCGGCCGCCGGGGGGGCGCCTTTGCCCCCC
GGGCCCGTGCCCNNNNNNNNNNGCCCGAAAGGCAGCGGCGGCACCGCGTCCGATCCTCGAGCGTATG
GGGCTTTGTCACATGCTCTGTAGGATTGGCCGGCGCCTGCCGACGTTTTCCAACCATTTTTTCCAGGTTG
ACCTCGGATCAGGTAGGGATACCCGCTGAACTTAA
>ASV65
TTTCCGTAGGTGAACCTGCGGAAGGATCATTACCGAGTGAGGGCCCTCTGGGTCCAACCTCCCACCCGT
GTTTATTTTACCTTGTTGCTTCGGCGGGCCCGCCTTAACTGGCCGCCGGGGGGCTCACGCCCCCGGGCC
CGCGCCCGCCGANNNNNNNNNNGGGCCCGAAAGGCAGCGGCGGCACCGCGTCCGGTCCTCGAGCGTA
TGGGGCTTTGTCACCCGCTCTGTAGGCCCGGCCGGCGCTTGCCGATCAACCCAAATTTTTATCCAGGTTG
ACCTCGGATCAGGTAGGGATACCCGCTGAACTTAA
>ASV67
TTTCCGTAGGTGAACCTGCGGAAGGATCATTATCGATGGCCGCGCCGTGAGCGCCCTAGTGCAGGATCG
GCCGGCCACTGACCACACCCTTACTTTACGAGCACCTTTCGTTCTCCTTCGGCGGGGCAACCCGCCGCT
GGAACCACATCANNNNNNNNNNGCGCGCGGACTCGCCCCAAAGGCATTGGCAGCGGTCCACGGCCCCT
CTCGCGCAGCACATTGCGCTTCTCGAGGCGGCCCGGCCCGCATCCACGAAGCCCACATTACCGTCTTTG
ACCTCGGATCAGGTAGGGATACCCGCTGAACTTAA
>ASV68
TTTCCGTAGGTGAACCTGCGGAAGGATCATTAGTGAATTGCTCTCTGAGCGTTAACTACATCCATCTACA
CCTGTGAACTGTTGATTGACTTCGGTCAATTGATTTTACAAACATTGTGTAATGAACGTCATGTTATTATA
ACAAAAATANNNNNNNNNNGGCTCGCCTTAAAAGAGTTAGCGTGTTTAACTTGTCTTATCTGGCGTAAT
AAGTTTCGCTGGTGTTGACTTGAGAAGTGCGCTTCTAATCGTCCTCGGACAATTCTTGAACTCTGGTCTC
AAATCAGGTAGGGCTACCCGCTGAACTTAA
>ASV72
TTTCCGTAGGTGAACCTGCGGAAGGATCATTAGTGATTTGCCTTCGGGCTAACTATATCCATAACACCTG
TGAACTGTTGATTGACTTCGGTCAATATTTTTACAAACATTGTGTAATGAACGTCATGTTATAATAACAA

ATATAACTTTNNNNNNNNNNCTCGTCTTAAAAGAGTTAGTGAATTTAACATTTGTCTTCTGGCGTAATA
AGTTTCGCTGGGCTGATAGTGTGAAGTTTGCTTCTAATCGTCCGCAAGGACAATTCTTGAACTCTGGCCT
CAAATCAGGTAGGACTACCCGCTGAACTTAA
>ASV75
TTTCCGTAGGTGAACCTGCGGAAGGATCATTACCGAGTGAGGGCCCTTTGGGTCCAACCTCCCACCCGT
GTTTATTTTACCTTGTTGCTTCGGCGGGCCCGCCTTTACTGGCCGCCGGGGGGCTTCACGCCCCCGGGC
CCGCGCCCGCCGNNNNNNNNNNGGCCCGAAAGGCAGCGGCGGCACCGCGTCCGGTCCTCGAGCGTA
TGGGGCTTTGTCACCCGCTCCGTAGGCCCGGCCGGCGCTTGCCGATCAACCCAAATTTTTATCCAGGTT
GACCTCGGATCAGGTAGGGATACCCGCTGAACTTAA
>ASV76
TCTCCGTAGGTGAACCTGCGGAGGGATCATTACACAATAACAAGGCGGGCTGGACACCCCCCGCTGGGC
ACTGCTTCACGGCGTGCGCGGCGGGGCCGGCCCTGCTGAATTATTCACCCGTGTCTTTTGCGTACTTCTT
GTTTCCTGGGTNNNNNNNNNNCTGGAGACTCGCCTTAAAGTCATTGGCAGCCGGCCTACTGGTTTCGG
AGCGCAGCACAAGTCGCGCTCTTCGCCAGCCAAGGTCAGCGTCCAGCAAGCCTTTTTTTCAACCTTTGA
CCTCGGATCAGGTAGGGATACCCGCTGAACTTAA
>ASV77
TATCTGTAGGTGAACCTGCAGATGGATCATTTCGATGAAAACCTTTTTTCTGAGGTGCGGCCCGCACCTG
TCTAACTAAACTAGGGCTACCCTTTTTCAACACGGTTGCATCGGTTGGGCTTGTCAGACAGCGCGCGCG
CAAGCGTGTTCNNNNNNNNNNCAGGACTTCGGAGGCGGAGAAAGAGCAAGAGCTGGACGCGACGACTT
TTGCTGGTTGGAGTGCTTCTGAACACCGCCCTTTTTTCTTTCTTGGAAAAAAGGAATTAATTCAAATCGG
CCTCAGATTGGTAGGACTACCCGCTGAACTTAA
>ASV83
TTTCCGTAGGTGAACCTGCGGAAGGATCATTAAGAATTATAAATATTTGTGAAATTTACACAGCAAACAA
TAATTTTATAGTCAAAACAAAAAAATCAAAACTTTTAACAATGGATCTCTTGGTTCTCGTATCGATGAAG
AACGCAGCGANNNNNNNNNNATTGCACTTTGGGGTATCCCCCAAAGTATACTTGTTTGAGCGTTGTTTC
TCTCTTGGAATTGCATTGCTTTTCTAAAATTTCGAATCAAATTCGTTTGAAAAACAACACTATTCAACCTC
AGATCAAGTAGGATTACCCGCTGAACTTAA
>ASV84
TTTCCGTAGGTGAACCTGCGGAAGGATCATTACCGAGTGAGGGCCCTTTGGGTCCAACCTCCCACCCGT
GTTTATTTTACCTTGTTGCTTCGGCGGGCCCGCCTTTACTGGCCGCCGGGGGGCTCACGCCCCCGGGCC
CGCGCCCGCCGANNNNNNNNNNGGCCCGAAAGGCAGCGGCGGCACCGCGTCCGGTCCTCGAGCGTA
TGGGGCTTTGTCACCCGCTCTGTAGGCCCGGCCGGCGCTTGCCGATCAACCCAAATTTTTATCCAGGTTG
ACCTCGGATCAGGTAGGGATACCCGCTGAACTTAA
>ASV90
TCTCCGTAGGTGAACCTGCGGAGGGATCATTACACAAATATGAAGGCGGGCTGGAACCTCTCGGGGTTA
CAGCCTTGCTGAATTATTCACCCTTGTCTTTTGCGTACTTCTTGTTTCCTTGGTGGGTTCGCCCACCACTA
GGACAAACATNNNNNNNNNNTGGAGACTCGCCTTAAAGTAATTGGCAGCCGGCCTACTGGTTTCGGAG
TGCAGCACAAGTCGCACTCTCTATCAGCAAAGGTCTAGCATCCATTAAGCCTTTTTTTCAACTTTTGACC
TCGGATCAGGTAGGGATACCCGCTGAACTTAA
>ASV91
TTTCCGTAGGTGAACCTGCGGAAGGATCATTAATAGTGCCCATTGATGCAAGTCATTGGGTTAGATCTGC
CCTTATCGCAAGATGAGGGCTTCCATATACACCGTGAACTGTGGCTTTGGCCATCACAAACTGTTAGTAA
TGAATGTAATNNNNNNNNNNGCGGTGGTGTATTGGGTGTTGCCTTGCTAAAGGCTCGCCTTAAAGACAT
AAGCACCTTGGATGTAATACGTTTCATCCTTCTGGGTGGCTGATAACCCCACATATTCATGATCTGGCCT
CAAATCAGGTAGGGCTACCCGCTGAACTTAA
>ASV92
TTTCCGTAGGTGAACCTGCGGAAGGATCATTATTGATTGGTCGAAAGACCTTATCAGATTCTACCACCTC
TGTGAACCGTTGACCTCCGGGTTAATAATCAAACATCAGTGTAACGAACGTAAGAGTATCTTAATTAAAC
AAAACTTTTANNNNNNNNNNGCTCACCTCAAATGACTTAGTGGAACATCCCACATCAGTGTTAGACGTA

ATAAGTTTCGTCTCTCCTTGTGGTGATGACTGCTCAAAACCTGCCATCGCGCACCTTTTGACTTTGACCT
GAAATCAGGTAGGGCTACCCGCTGAACTTAA
>ASV93
TTTCCGTAGGTGAACCTGCGGAAGGATCATTAAAAAAACACTTATACACTTTTAGGCATAAACACTTACC
TTTGAATTCTTCAAATACACAATTAAAAAACTTTCAACAACGGATCTCTTGGTTCTCGCATCGATGAAGA
ACGCAGCGAANNNNNNNNNNCCGGGGTATTCCCCAGGGCATGCGTGGGTGAGCGATATTTACTCTCAA
ACCTCCGGTTTGGTCCTGCTTCGGCATAATATCAACGGCGCTAGAATAAGTTTTAGCCCCAGCCTTTTTC
CTCACCCTCGTAAGACTACCCGCTGAACTTAA
>ASV95
TTTCCGTAGGTGAACCTGCGGAAGGATCATTATGAATTAATAATATTTGTGAATTTACCACAGCAAACAA
AAATCATACAATCAAAACAAAAATAATTAAAACTTTTAACAACGGATCTCTTGGTTCTCGTATCGATGAA
GAACGCAGCGNNNNNNNNNNATTGCACTTTGGGGTATCCCCCAAAGTATACTTGTTTGAGCGTTGTTTC
TCTCTTGGAATTGCTTTGCTCTTCTAAAATTTCGAATCAAATTCGTTTGAAAAACAACACTATTCAACCTC
AGATCAAGTAGGATTACCCGCTGAACTTAA
>ASV96
TTTCCGTAGGTGAACCTGCGGAAGGATCATTAATAAAAATACTAAACACTGTTCTTTTTTAAAAAAAACC
ATTTACTAATTCTAATTTTTATATTAAAACTTTCAACAACGGATCTCTTGGTTCTCGCATCGATGAAGAAC
GCAGCGAAANNNNNNNNNNGCACCGTGGGGTATTCCCCACGGTATGCATTGATGAGCGGTAAACTCCC
TCGTCTACGACGGTCTTGCCACTAAAGAAAAAAGTTCAATATCAAAAACATTGTCCTCTTCTCCTTTCCC
TCATCAATGTAAGACTACCCGCTGAACTTAA
>ASV98
TTTCCGTAGGTGAACCTGCGGAAGGATCATTAGTGATTGCCATCTTGGCTTAAAACTATATCCACATACA
CCTGTGCACTGTTCGATTGACTCTCACGAGTCAGTTTTTACAAACATTGTGTAATGAACGTCTAGTTATT
ATAACAAAAANNNNNNNNNNTGGCTCGCCTTAAAAGAGTTAGCAAACATAACATTGTTGTCTGGTGTAA
TAAGTTTCACTGGTAAGACGTGTGACTGTAAGCTTCTAATCGCCCTTGGGCAATTTTTTGACTCTGGCCT
CAAATCAGGTAGGACTACCCGCTGAACTTAA
>ASV99
TTTCCGTAGGTGAACCTGCGGAAGGATCATTATGAATTAATAATATTTGTGAATTTACCACAGCAAACAA
AAATCATACAATCAAAACAAAAATAATTAAAACTTTTAACAATGGATCTCTTGGTTCTCGTATCGATGAA
GAACGCAGCGNNNNNNNNNNATTGCACTTTGGGGTATCCCCCAAAGTATACTTGTTTGAGCGTTGTTTC
TCTCTTGGAATTGCTTTGCTCTTCTAAAATTTCGAATCAAATTCGTTTGAAGAACAACACTATTCAACCTC
AGATCAAGTAGGATTACCCGCTGAACTTAA
>ASV101
TCTCCGTAGGTGAACCTGCGGAGGGATCATTACACAAATATGAAGGCGGGCTGGAACCTCTCGGGGTTA
CAGCCTTGCTGAATTATTCACCCTTGTCTTTTGCGTACTTCTTGTTTCCTTGGTGGGTTCGCCCACCACTA
GGACAAACATNNNNNNNNNNCTGGAGACTCGCCTTAAAGTAATTGGCAGCCGGCCTACTGGTTTCGGA
GCGCAGCACAAGTCGCACTCTCTATCAGCAAAGGTCTAGCATCCATTAAGCCTTTTTTCAACTTTTGACC
TCGGATCAGGTAGGGATACCCGCTGAACTTAA
>ASV102
TTTCCGTAGGTGAACCTGCGGAAGGATCATTACCGAGTGTTTGGATGCCCCTTCTCGGGGTGTCCGTCCT
CCCATCCGTGTCTATTTGTACCCTGTTGCTTCGGCGGGCCCGCCCTTCGTGGCCGCCGGGGGGCTTCCC
TGCCCCCGGGCNNNNNNNNNNGGCAGCGGCGGCACCGTGTCCGGTCCTCGAGCGTATGGGAAGCAACT
TTTTGTCACCCGCTCCTGTAGGTCCGGCCGGCGGCCTGCCCAACCCCAACCTTTTTTTAACCAGGTTGAC
CTCGGATCAGGTAGGGATACCCGCTGAACTTAA
>ASV115
TTTCCGTAGGTGAACCTGCGGAAGGATCATTATAGAAATATAATTATTCGTTGCTTTGCTTGGAGACACA
TACTGCCGAACCAGCGCTTAATTGCGCGGTTTGGTGGGTCTCTGTAGCTCAGTAGCACTATTACACACTG
TGGAGATTTTNNNNNNNNNNGCTGGACATCTTCGTACTAGGTTTTACCAATTCGAGGACGGTTAGCGAG
GCGGCCTGCAGTGAGTGTAGTGCTTGACTACGTTGCACCATGGCGAACAGTGTTCTTTTAAGTTTGACCT
CAAATCAGGTAGGAGTACCCGCTGAACTTAA

>ASV116
TTTCCGTAGGTGAACCTGCGGAAGGATCATTACTGAGTGAGGGCCCTCTGGGTCCAACCTCCCACCCGT
GTTTATTGTACCTTGTTGCTTCGGTGCGCCCGCCTCACGGCCGCCGGGGGGCTTCTGCCCCCGGGTCCG
CGCGCACCGGAGNNNNNNNNNNGGTCCGAAAGGCAGCGGCGGCACCGAGTCCGGTCCTCGAGCGTAT
GGGGCTTTGTCACCCGCTCTGTAGGCCCGGCCGGCGCCAGCCGACAACCAATCATCCTTTTTTCAGGTT
GACCTCGGATCAGGTAGGGATACCCGCTGAACTTAA
>ASV117
TTTCCGTAGGTGAACCTGCGGAAGGATCATTACCGTGGGGCTTCGGCTCCGTCGAGATAATACCCTTGC
CTTTTTGAGTACCAACCGTTTCCTCGGCAGGTCCGCCTGCCAATGAGGACCCCAAACCAAACCTTTTGTC
ACCTGTATTAANNNNNNNNNNCGCGCGTGGACTCACCTCAAATCTATTGGCGGCCCTCGTGCCGGCCC
CGAGCGCAGCAGAAACGCGGTCTCGTGGCCCGGCGGAGGTGCCCCCAGAAGCAACATTCACCGTTTTGA
CCTCGGATCAGGTAGGGATACCCGCTGAACTTAA
>ASV118
TCTCCGTTGGTGAACCAGCGGAGGGATCATTACCGAGTTTACAACTCCCAAACCCCTGTGAACATACCTT
TATGTTGCCTCGGCGGATCAGCCCGCGCCCCGTAAAACGGGACGGCCCGCCGCAGGAAACCCTAAACTC
TGTTTTTAGTGNNNNNNNNNNAACACAGTCCCCAAATTGATTGGCGGTCACGTCGAGCTTCCATAGCGT
AGTAATTTACACATCGTTACTGGTAATCGTCGCGGCCACGCCGTTAAACCCCAACTTCTGAATGTTGACC
TCGGATCAGGTAGGAATACCCGCTGAACTTAA
>ASV119
TTTCCGTAGGTGAACCTGCGGAAGGATCATTAGTGATAAAACTATTATCTTAACACCTGTGAACTGTGAA
CCGAAAGGTTCTTCCAAACATTGTGTAATGAACGTAATACATTATAAACAATACAACTTTCAACAACGGA
TCTCTTGGCTNNNNNNNNNNATCGCTCACCTTAAAGGAGTTAGCAACTAAGCGATGTCGTCCGACGTAA
TAAGTTTCGTCTGGTAATTCGACTGAGCCAATTGCTTCTAATTGTCTTTTGACTTTTTTGACTCTGGCCTC
AAATCAGGTAGGACTACCCGCTGAACTTAA
>ASV123
TTTCCGTAGGTGAACCTGCGGAAGGATCATTACAGTATTCTTTTGCCAGCGCTTAATTGCGCGGCGAAAA
ACCTTACACACTATGTTTTTTTGATTTGAAACTTTTGCTTTGGTCTGACTTAGAAATGAGTTGGGCCAAA
GGTTTTATACNNNNNNNNNNGAGTGGTACTAGATAGTGCTGAACTGTTTCAATGTATTAGGTTTATCCA
ACTCGTTGACCAGTATAGTATTTGTTTATTACACAGGCTCGGCCTTACAACAACAAACAAAGTTTGACCT
CAAATCAGGTAGGACTACCCGCTGAACTTAA
>ASV124
TTTCCGTAGGTGAACCTGCGGAAGGATCATTACAGAGTTCATGCCCTCACGGGTAGATCTCCCACCCTTG
AATACTATACCTTAGTTGCTTTGGCAGGCCGTGGAAACACCCTGGGCTCCGGCTCGGGCGTGCCTGCCA
GAGGAAACAAANNNNNNNNNNGTTTCGCAGCCTCTAAACCCAGTGGCGGTGCCATTGAGCTCTGAGCG
TAGTAATTATCCTCGCTATAGAGTCTCGGTGGTGTCTTGCCAGCAACCCCTAATATTTTTACAGGTTGAC
CTCGGATCAGGTAGGGATACCCGCTGAACTTAA
>ASV125
TCTCCGTTGGTGAACCAGCGGAGGGATCATTACCGAGTTTACAACTCCCAAACCCCTGTGAACATACCA
TATGTTGCCTCGGCGGATCAGCCCGTCCTTCGGGACGGCCCGCCGCAGGACCCTAAACTCTGTTTTTAG
TGGAACTTCTGANNNNNNNNNNTTCGCATTCCCCAAATTGATTGGCGGTCACGTCGAGCTTCCATAGCG
TAGTAATTTACACATCGTTACTGGTAATCGTCGCGGCCACGCCGTTAAACCCCAACTTCTGAATGTTGAC
CTCGGATCAGGTAGGAATACCCGCTGAACTTAA
>ASV126
TTTCCGTAGGTGAACCTGCGGAAGGATCATTACCGAGTGCGGGCCCTCTGGGTCCAACCTCCCATCCGT
GTCTATCTGTACCCTGTTGCTTCGGCGTGGCCACGGCCCGCCGAAGACTAACATTTGAACACTGTCTGAA
GTTTGCAGTCTNNNNNNNNNNGCCCAAAAGGCAGTGGCGGCACCATGTCTGGTCCTCGAGCGTATGGG
GCTTTGTCACCCGCTCCCGTAGGTCCAGCTGGCAGCTAGCCTCGCAACCAATCTTTTTAACCAGGTTGAC
CTCGGATCAGGTAGGGATACCCGCTGAACTTAA
>ASV127

TTTCCGTAGGTGAACCTGCGGAAGGATCATTAAATAATCAATAATTTTGGCTTGTCCATTATTATCTATTT
ACTGTGAACTGTATTATTACTTGACGCTTGAGGGATGCTCCACTGCTATAAGGATAGGCGATGGAGATGC
TAACCGAGTNNNNNNNNNNNTGAAATGTACAAAGGCCTGATCTTGTTTGAATGCCTGAACTTTTTTTTAA
TATAAAGAGAAGCTCTTGCGGTAAACTGTGCTGGGGCCTCCCAAATAATACTTTTTTTAAATTTGATCTG
AAATCAGGCGGGATTACCCGCTGAACTTAA
>ASV128
TTTCCGTAGGTGAACCTGCGGAAGGATCATTAAAGAGACGTTGCCCTTCGGGGTATACCTCCCACCCTTT
GTATATCATACCTTCAAACTCTGTTATTAGTGTCGTCTGAGTACTATAAACAATAGTTAAAACTTTCAACA
ACGGATCTCNNNNNNNNNNNTTCCGGCAGGCCCCAAAATCAGTGGCGGCGCCATTCGGCTTCAAGCGTA
GTAACTACTTCTCGCTTCGGAGGACCGGGTGCGTGCTCGCCAGCAACCCCCAATTTATTCAGGTTGACCT
CGGATCAGGTAGGGATACCCGCTGAACTTAA
>ASV136
TTTCCGTAGGTGAACCTGCGGAAGGATCATTACAACAAGGTTCCCTGGCCCTCGAAGCTTCGGCGGAGG
GGTGCTACAGCCTGACTTTATACCCACCCTTTGCCTATGTGTACCCCTATTGCTTCCCTCGGCGGGTTCG
CCCGCCGACAGNNNNNNNNNNNGTGCGCGGACTCGCCTCAAAGTCATTGGCAGCGGTCTCGTCGGCTTC
TCGCGCAGCACATTTGCGCTTCTTGGAGCCCCGGCGGATCAGCGTCCAGCAAGCAATTTCATGACTTGA
CCTCGGATCAGGTAGGGATACCCGCTGAACTTAA
>ASV138
TTTCCGTAGGTGAACCTGCGGAAGGATCATTATAGAAATATAATTTTTCGTTGCTTTGCGAGGAGACACT
ATACTGCTGGACCAGCGCTTAATTGCGCGGTTTGGTGGGTCTCTGTAGCTCAGTAGCACTATTACACACA
GTGGAGATTTNNNNNNNNNNNTGGACATCTTCGTATTAGGTTCTACCAACTTCGAAGACGGTTAGCGGGG
AGTTCTGCAGTGAGTGTAGTGCTTTTACTACGTTGCACCATGGCGAACAGTGTTCTTTTAAGTTTGACCT
CAAATCAGGTAGGATTACCCGCTGAACTTAA
>ASV144
TTTCCGTAGGTGAACCTGCGGAAGGATCATTATGAATTAATAATATTTGTGAATTTACCACAGCAAACAA
AAACCATACAATCAAAACAAAAATAATTAAAACTTTTAACAATGGATCTCTTGGTTCTCGTATCGATGAA
GAACGCAGCGNNNNNNNNNNNATTGCACTTTGGGGTATCCCCCAAAGTATACTTGTTTGAGCGTTGTTTC
TCTCTTGGAATTGCTTTGCTCTTCTAAAATTTCGAATCAAATTCGTTTGAAAAACAACACTATTCAACCTC
AGATCAAGTAGGATTACCCGCTGAACTTAA
>ASV147
TTTCCGTAGGTGAACCTGCGGAAGGATCATTATGAATTAATAATATTTGTGAATTTACCACAGCAAACAA
AAATCATACAATCAAAACAAAAATAATTAAAACTTTTAACAATGGATCTCTTGGTTCTCGTATCGATGAA
GAACGCAGCGNNNNNNNNNNNATTGCACTTTGGGGTATCCCCCAAAGTATACTTGTTTGAGCGTTGTTTC
TCTCTTGGAATTGCTTTGCTCTTCTAAAATTTCGAATCAAATTCGTTTGAAAAACAACACTATCCAACCTC
AGATCAAGTAGGATTACCCGCTGAACTTAA
>ASV151
TTTCCGTAGGTGAACCTGCGGAAGGATCATTAATGATTGAACGTCTGTCGAGCTTGCTCACAGGCACATC
ATATCCATAACACCTGTGCACTTGTCGGATGGCTTAGTGAAGACCGCAAGGTTGGATCTATCCATCTACT
TTACATAACANNNNNNNNNNNTGGTTTGACTTGGCGTAATAAGTATTTCGCTAAGGACATCTTCGGATGG
CCGCGTTGCAGGACTAAAGACCGCTTTCTAATCCATTGATCTTACGATTAATATTCTTGACATCTGGCCT
CAAATCAGGTAGGACTACCCGCTGAACTTAA
>ASV156
TTTCCGTAGGTGAACCTGCGGAAGGATCATTAATGAAAATGTGTTGCCGGGGCCCATAATCCCGGCACT
AACCTTCTTATCCATAACACCTGTGCACTGTTGGATGCTTGCATCCACTTTTATACTAAACAATTTGTAAC
AAATGTAGTCNNNNNNNNNNNAATAAGTATTTTGCTAAGGACATCTTCGGATGGCCAGGACTTGACTTTT
GTCTGCTTACTAAACCTTACTTTAAGTGCATCTCTGGTGTTACTTATAGTATTACTTTGACATATGGCCTC
AAATCAGGTAGGACTACCCGCTGAACTTAA
>ASV159
TTTCCGTAGGTGAACCTGCGGAAGGATCATTATGAATTAATAATATTTGTGAATTTACCACAGCAAACAA
AAATCATAAAATCAAAACAAAAATAATTAAAACTTTTAACAATGGATCTCTTGGTTCTCGTATCGATGAA

GAACGCAGCGNNNNNNNNNNATTGCACTTTGGGGTATCCCCCAAAGTATACTTGTTTGAGCGTTGTTTC
TCTCTTGGAATTGCTTTGCTCTTCTAAAATTTCGAATCAAATTCGTTTGAAAAACAACACTATTCAACCTC
AGATCAAGTAGGATTACCCGCTGAACTTAA
>ASV160
TTTCCGTAGGTGAACCTGCGGAAGGATCATTATAATTGTTTTTACACCCTTTTAGGCACAAACTCTAAAT
CTTAACCCTCAATAACTTTATTAAAAAACTTTCAACAACGGATCTCTTGGTTCTCGCATCGATGAAGAAC
GCAGCGAATTNNNNNNNNNNCCGGGGTATTCCCCAGGGCATGCGTGGGTGAGCGATATTTACTCTCAA
ACCTCTGGTTTGGTCCTGCTTCGGCCTAATATCAACGGCGCTAGAATAAGTTTTAGCCCCATTCTTCTTC
CTCACCCTCGTAAGACTACCCGCTGAACTTAA
>ASV168
TTTCCGTAGGTGAACCTGCGGAAGGATCATTATGAATTAATAATATTTGTGAAATTTCAACAAACAACAT
CAATTTTATAGTCTATCATTTTTAATTAAAACTTTTAACAATGGATCTCTTGGTTCTCGTATCGATGAAGA
ACGCAGCGANNNNNNNNNNATTGCACTTTGGGGTATCCCCCAAAGTATACTTGTTTGAGCGTTGTTTCT
CTCTTGGAATTGCATTGCTTTTCTAAAAAATCGAATCAAATTCGTTTGAAAAACAACACTATTCAACCTC
AGATCAAGTAGGATTACCCGCTGAACTTAA
>ASV169
TTTCCGTAGGTGAACCTGCGGAAGGATCATTATGAATTAATAATATTTGTGAATTTACCACAGCAAACAA
AAATCATACAATCAAAACAAAAATAATTAAAACTTTTAACAATGGATCTCTTGGTTCTCGTATCGATGAA
GAACGCGGCGNNNNNNNNNNATTGCACTTTGGGGTATCCCCCAAAGTATACTTGTTTGAGCGTTGTTTC
TCTCTTGGAATTGCTTTGCTCTTCTAAAATTTCGAATCAAATTCGTTTGAAAAACAACACTATTCAACCTC
AGATCAAGTAGGATTACCCGCTGAACTTAA
>ASV173
TTTCCGTAGGTGAACCTGCGGAAGGATCATTACCGAGTGCGGGCTGCCTCCGGGCGCCCAACCTCCCAC
CCGTGACTACCTAACACTGTTGCTTCGGCGGGGAGCCCTCTCGGGGGCGAGCCGCCGGGGACTACTGAA
CTTCATGCCTGANNNNNNNNNNCCCGAAAGGCAGCGGCGGCACCGTGTCCGGTCCTCGAGCGTATGGG
GCTTTGTCACCCGCTCGATTAGGGCCGGCCGGGCGCCAGCCGACGTCTCCAACCATTTTTTTCAGGTTG
ACCTCGGATCAGGTAGGGATACCCGCTGAACTTAA
>ASV174
TCTCCGTTGGTGAACCAGCGGAGGGATCATTACAGAGTTATCCAACTCCCAAACCCATGTGAACTTATCT
CTTTGTTGCCTCGGCGCAAGCTACCCGGGACCCAGCGCCCCGGGCGGCCCGCCGGCGGACAAACCAAA
CTCTTGTTATCTNNNNNNNNNNAGTTCCCTAATGCGATTGGCGGAGTGGCAGTAGTCCTCTGAGCGTAG
TAATTCTTTATCTCGCTTTTGTTAGGTGCTGCCCCCCCGGCCGTTAAACCCCCAATTTTTTCTGGTTGACC
TCGGATCAGGTAGGAATACCCGCTGAACTTAA
>ASV175
TTTCCGTAGGTGAACCTGCGGAAGGATCATTATGAATTAATAATATTTGTGAATTTACCACAGCAAACAA
AAATCATACAATCAAAACAAAAATAATTAAAACTTTTAACAATGGATCTCTTGGTTCTCGTATCGATGAA
GAACACAGCGNNNNNNNNNNATTGCACTTTGGGGTATCCCCCAAAGTATACTTGTTTGAGCGTTGTTTC
TCTCTTGGAATTGCTTTGCTCTTCTAAAATTTCGAATCAAATTCGTTTGAAAAACAACACTATTCAACCTC
AGATCAAGTAGGATTACCCGCTGAACTTAA
>ASV183
TCTCCGTTGGTGTACCAGCGGAGGGATCATTACCGAGTTTACAACTCCCAAACCCATGTGAACATACCTT
ACAGTTGCTTCGGCGGAGCCGCCCCGGCGCCCGGAACCCAGTTTCGCGGCCCGGACCAAGGCGCCCGC
CGGAGGCCACAANNNNNNNNNNCCGCCGGCCCCGAAATGAAGTGGCGGCCCGTCCGCGGCGACCTCT
GCGTAGTAACTCCACTCGCACCGGGACCCGGGCGCGGCCACGCCGTAAAACCCCCAACTTCCGAATGTT
GACCTCGAATCAGGTAGGAATACCCGCTGAACTTAA
>ASV185
TTTCCGTAGGTGAACCTGCGGAAGGATCATTACCGAGTGCGGGCTGCCTCCGGGCGCCCAACCTCCCAC
CCGTGACTACCTAACACTGTTGCTTCGGCGGGGAGCCCTTTCGGGGGCGAGCCGCCGGGGACTACTGAA
CTTCATGCCTGANNNNNNNNNNCCCGAAAGGCAGCGGCGGCACCGTGTCCGGTCCTCGAGCGTATGGG

GCTTTGTCACCCGCTCGATTAGGGCCGGCCGGGCGCCAGCCGACGTCTCCAACCATTTTTTTCAGGTTG
ACCTCGGATCAGGTAGGGATACCCGCTGAACTTAA
>ASV186
TTTCCGTAGGTGAACCTGCGGAAGGATCATTACCGAGTGAGGGCCCTCTGGGTCCAACCTCCCACCCGT
GTCTATCGTACCTTGTTGCTTCGGCGGGCCCGCCGTTTCGACGGCCGCCGGGGAGGCCTTGCGCCCCCG
GGCCCGCGCCCGNNNNNNNNNNGGCCCGAAAGGCAGCGGCGGCACCGCGTCCGGTCCTCGAGCGTAT
GGGGCTTTGTCACCTGCTCTGTAGGCCCGGCCGGCGCCAGCCGACACCCAACTTTATTTTTCTAAGGTTG
ACCTCGGATCAGGTAGGGATACCCGCTGAACTTAA
>ASV188
TTTCCGTAGGTGAACCTGCGGAAGGATCATTATGAATTAATAATATTTGTGAATTTACCACAGCAAACAA
AAATCATACAATCAAAACAAAAATAATTAAAACTTTTAACAATGGATCTCTTGGTTCTCGTATCGATGAA
GAACGCAGCGNNNNNNNNNNATTGCACTTTGGGGTATCCCCCAAAGTATACTTGTTTGAGCGTTGTTTC
TCTCTTGGAATTGCTTTGCTCTTCTAAAATTTCGAATCAAATTCGTTTGAAAAACAACACTATTCAACCTC
AGATCGAGTAGGATTACCCGCTGAACTTAA
>ASV191
TTTCTGTAGGTGAACCTGCAGAAGGATCATTAGTGAAGATTTGGGCAGGCCATACGGACGCCAAAAAGT
GTCCCTGGCCGCCTACACCCACTATACATCCACAAACCCGTGTGCACTGTCTTGGAGAAAGGCTTCAGA
GAAGTTTTTTGTNNNNNNNNNNTTTCTCCTGGCATGGCATGATACGTCATTTGCTATGTCGCCTAAAGG
AGGAATGTTTGGTTGTGTCTACGTGTGCTTCGAACTTGCCTCTGTGGCACATCCCAATTTCACTTCTGGT
CTCAAATCAGGTAGGATCACCCGCTGAACTTAA
>ASV210
TTTCCGTAGGTGAACCTGCGGAAGGATCATTACAGAGTTCATGCCCTTACGGGTAGATCTCCCACCCTTG
AGTATTATACTTAGTTGCTTTGGCAGGCTGCTTCGGCAGCTGGCTTCGGCCAGTCACGCCTGCCAAAGG
ATACCTAAACTNNNNNNNNNNGCCTATCGGCAGCCCTTAAAATCAGTGGCGGTGCTATTTTGCTCTGAG
CGTAGTAAATCTTCTCGCTATAGAGTCTTGGTAGCCACTTGCCAACAACCTAAACTTCTATGGTTTGACC
TCGGATCAGGTAGGGATACCCGCTGAACTTAA
>ASV212
TCTCCGTAGGTGAACCTGCGGAGGGATCATTACAAGAACGCCCGGGCTTCGGCCTGGTTATTCATAACC
CTTTGTTGTCCGACTCTGTTGCCTCCGGGGCGACCCTGCCTTCGGGCGGGGGCTCCGGGTGGACACTTC
AAACTCTTGCGTNNNNNNNNNNCGCCGCGTGCCTCAAATCGACCGGCTGGGTCTTCTGTCCCCTAAGC
GTTGTGGAAACTATTCGCTAAAGGGTGTTCGGGAGGCTACGCCGTAAAACAACCCCATTTCTAAGGTTG
ACCTCGGATCAGGTAGGGATACCCGCTGAACTTAA
>ASV213
TTTCCGTAGGTGAACCTGCGGAAGGATCATTAATGAATTTTAGGACGTTCTTTTTAGAAGTCCGACCCTT
TCATTTTCTTACACCGTGCACACACTTCTTTTTTACACACACTTTTAACACCTTAGTATAAGAATGTAATA
GTCTCTTAANNNNNNNNNNAGCAACCCTACTGAAATAAACGGTTTGACTTGGCGTAATAATTATTTCGC
TAAGGACGTTTTCTTCAATTATAAGAGGTGCTTCTAATTCGCTTCTAATAGCATTTAAGCTTTAGACCTCA
AATCAGTCAGGACTACCCGCTGAACTTAA
>ASV215
TCTCCGTTGGTGAACCAGCGGAGGGATCATTACCGAGTACTATAACTCATAACCCTTTGTGAACCTTTAT
ACCTGTTGCTTCGGCGGCGCGCCTCCCGGGGCGTGCCCGCCGGCATTATCAGAATCTCTGTTCGAACCC
GACGATACATCNNNNNNNNNNGTGGCGGACCCGTTGGGCCCTTCCTTTGCGTAGTAACATCTGCCTCG
CATCGGGAGCCTGCGGGCTATCCGGCCTCTAAACCCCCCTCAAGCCCGCTCCGGCGGCACCAAGGTTGA
CCTCGGATCAGGTAGGAATACCCGCTGAACTTAA
>ASV222
TTTCCGTAGGTGAACCTGCGGAAGGATCATTACCGAGTGCGGGTTCCAACGAGCCCAACCTCCCACCCG
TGTTTACCATGACCGCGTTGCCTCGGCGGGCCCACTGGGGCCTGGCCCCGGTCGCCGGGGGGCTCCTGC
CCCCGGGTCCGCNNNNNNNNNNTGGCGGCGCCGCGTCGGGTCCTCGAGCGTATGGGGCTTTGTCACCC
GCTCGGGAAGGACTCGTCGGCGCTGGTCTTCCTCCAGGCGACCCTTCGGGGCTCGTCTTCTTTCGGTTG
ACCTCGGATCAGGTAGGGTTACCCGCTGAACTTAA

>ASV226
TTTCCGTAGGTGAACCTGCGGAAGGATCATTATCCATCTCAAACCAGGTGCGGTCGCGGCCCCCGGGGG
CTTGCTCCCGGGTGGTAGGGGTAACACCCTCACGCGCCGCCTGCCTGTACCCTCTTTTTACGAGCACCTT
TCGTTCTCCTTNNNNNNNNNNTGCGCGCGGACTCGCCCCAAATTCATTGGCAGCGGTCTTTGCCTCCTC
TCGCGCAGCACAATTGCGTCTGCGGGGGGGCGTGACCCGCGTCCACGAAGCAACATTACCGTCTTTGAC
CTCGGATCAGGTAGGGATACCCGCTGAACTTAA
>ASV231
TATCTGTAGGTGAACCTGCAGATGGATCATTTCGATGAAAACCTTTTTTCTGAGGTGCGGCCCGCACCTG
TCTAACTAAACTAGGGCTACCCTTTTTCAACACGGTTGCATCGGTTGGGCTTGTCAGACAGCGCGCGCG
CAAGCGTGTTCNNNNNNNNNNTCAGGACTTCGGAGGCGGAGAAAGAGCAAGAGCTGGACGCGACGACT
TTTGCTGGTTGGAGTGCTTCTGAACACCGCCCTTTTTTCTTTCTTGAAAAAAGGAATTAATTCAAATCGG
CCTCAGATTGGTAGGACTACCCGCTGAACTTAA
>ASV233
TTTCCGTAGGTGAACCTGCGGAAGGATCATTATGAATTAATAATATTTGTGAATTTACCACAGCAAACAA
AAAGCATACAATCAAAACAAAAATAATTAAAACTTTTAACAATGGATCTCTTGGTTCTCGTATCGATGAA
GAACGCAGCGNNNNNNNNNNATTGCACTTTGGGGTATCCCCCAAAGTATACTTGTTTGAGCGTTGTTTC
TCTCTTGGAATTGCTTTGCTCTTCTAAAATTTCGAATCAAATTCGTTTGAAAAACAACACTATTCAACCTC
AGATCAAGTAGGATTACCCGCTGAACTTAA
>ASV234
CGTTTGCTTGAAATGTATTGGCATGAGTGGTACTGGATAGTGCTATATGACTTTCAATGTATTAGGTTTA
TCCAACTCGTTGAATAGTTTAATGGTATATTTCTCGGTATTCTAGGCTCGGCCTTACAATATAACAAACA
AGTTTGACCTNNNNNNNNNNACTGGATAGTGCTATATGACTTTCAATGTATTAGGTTTATCCAACTCGT
TGAATAGTTTAATGGTATATTTCTCGGTATTCTAGGCTCGGCCTTACAATATAACAAACAAGTTTGACCT
CAAATCAGGTAGGACTACCCGCTGAACTTAA
>ASV236
TTTCCGTAGGTGAACCTGCGGAAGGATCATTAGTGATTGCCTTTATTGGCTTATAACTATATCCATCTAC
ACCTGTGAACTGTTCGATTGAATCATTAATTTGATTCAATTTTTTACAAACATTGTGTAATGAACGTCATT
AGATCATAANNNNNNNNNNCTCCTCTTAAAGGAGTTAGCAAGTTGAACTATTGCTATCTGGCGTAATAA
GTTTCGCTGGAATTAGTATTGTGAAGCGTGCTTCTAATCGTCTTCGGACAATTATTTTGACTCTGGCCTC
AAATCAGGTAGGACTACCCGCTGAACTTAA
>ASV239
TTTCCGTAGGTGAACCTGCGGAAGGATCATTACCGAGTGAGGGCCCTCTGGGTCCAACCTCCCACCCGT
GTTTATTTACCTTATTGCTTCGGCGGGCCCGCCTTAACTGGCCGCCGGGGGGTTTACACCCCCGGGCCC
GCGCCCGCCGAANNNNNNNNNNGGGCCCGAAAGGCAGCGGCGGCACCGCGTCCGGTCCTCGAGCGTA
TGGGGCTTTGTCACCCGCTCTGTAGGCCCGGCCGGCGCTTGCCGATCAACCCAAATTTTTATCCAGGTTG
ACCTCGGATCAGGTAGGGATACCCGCTGAACTTAA
>ASV242
TTTCCGTAGGTGAACCTGCGGAAGGATCATTAGTGATTTGCCTTCGGGCTAAACTATATCCATAACACCT
GTGAACTGTTGATTGACTTCGGTCAATATTTTTACAAACATTGTGTAATGAACGTCATGTTATAATAACA
AATATAACTTNNNNNNNNNNCTCGTCTTAAAAGAGTTAGTGAATTTAACATTTGTCTTCTGGCGTAATA
AGTTTCGCTGGGCTGATAGTGTGAAGTTTGCTTCTAATCGTCCGCAAGGACAATTCTTGAACTCTGGCCT
CAAATCAGGTAGGACTACCCGCTGAACTTAA
>ASV249
TTTCCGTAGGTGAACCTGCGGAAGGATCATTAATGAATGAAATTGAGTGGGGTTGTAGCTGGCCTTTTAC
CGGGCATGTGCACACCTCATTCAACCACCTTCTATACCTCTGTGCACTTCTCATGAGTTGGGCCGCGTCT
GAAATATGACNNNNNNNNNNTTAGCTTGAATAGAACCAAGCATGATTCAGCGTGATAATTGTCTACGTT
GCTTCATCTCGGTGAATTTAATGTTCGAGCTTCTAACCGTCCCTCGGGACAATATCTGAACATCTGACCT
CAAATCAAGTAGGACTACCCGCTGAACTTAA
>ASV251

TTTCCGTAGGTGAACCTGCGGAAGGATCATTACAGAGTTCATGCCCGAAAGGGTAGACCTCCCACCCTT
GTGTATTATTACTTTGTTGCTTTGGCGAGCTGCCTTCGGGCCTTGTATGCTCGCCAGAGAATACCAAAAC
TCTTTTTATTANNNNNNNNNNCAGTAATGGCAGGCTCTAAAATCAGTGGCGGCGCCGCTGGGTCCTGAA
CGTAGTAATATCTCTCGTTACAGGTTCTCGGTGTGCTTCTGCCAAAACCCAAATTTTTCTATGGTTGACC
TCGGATCAGGTAGGGATACCCGCTGAACTTAA
>ASV254
CGTTTGCTTGAAATGTATTGGCATGAGTGGTACTGGATAGTGCTATATGACTTTCAATGTATTAGGTTTA
TCCAACTCGTTGAATAGTTTAATGGTATATTTCTCGGTATTCTAGGCTCGGCCTTACAATATAACAAACA
AGTTTGACCTNNNNNNNNNNACTGGATAGTGCTATATGACTTTCAATGTATTAGGTTTATCCAACTCGT
TGAATAGTTTAATGGTATATTTCTCGGTATTCTAGGCTCGGCCTTACAATATAACAAACAAGTTTGACCT
CAAATCAGGTAGGATTACCCGCTGAACTTAA
>ASV255
TTTCCGTAGGTGAACCTGCGGAAGGATCATTAGTGATTGCCTTTATAGGCTTAACTATATCCACATACAC
CTGTGAACTGTTCTACCACTTGACGCAAGTCGAGTGTTTTTACAAACAATGTGTAATGAACGTCGTTTTA
TTATAACAAANNNNNNNNNNTCGCCTTAAAAGAGTTAGCAAGTTTGACATTAATGTCTGGTGTAATAAG
TTTCACTGGGTCCATTGTGTTGAAGCGTGCTTCTAATCGTCCGCAAGGACAATTACTTTGACTCTGGCCT
GAAATCAGGTAGGACTACCCGCTGAACTTAA
>ASV256
TTTCCGTAGGTGAACCTGCGGAAGGATCATTACCGAGTGCTGGGTCCTTCGGGGCCCAACCTCCCACCC
GTGCTTACCGTACCCTGTTGCTTCGGCGGGCCCGCCTTCGGGCGGCCCGGGGCCTGCCCCCGGGACCGC
GCCCGCCGGAGANNNNNNNNNNGGGGCGGGCCTCGAGAGAAACGGCGGCACCGTCCGGTCCTCGAGC
GTATGGGGCTCTGTCACCCGCTCTATGGGCCCGGCCGGGGCTTGCCTCGACCCCCAATCTTCTCAGATT
GACCTCGGATCAGGTAGGGATACCCGCTGAACTTAA
>ASV258
TTTCCGTAGGTGAACCTGCGGAAGGATCATTACACTCAGTAGTTTACTACTGTAAAGGAGGCTGTTAGTC
TGTATAGCGCAAGCTGATGAGCAGCTAGCCTCTTTTATCCACCCTTGTCTTTTGCGTACCCACGTTTCCT
CGGCAGGCTTNNNNNNNNNNCTAGTGTTTGGACTCGCCTTAAAATAATTGGCAGCCAGTGTTTTGGTAT
TGAAGCGCAGCACAAGTCGCGATTCTTATCAAATACTTGCGTCCACAAGCCCTTTTTTAACTTTTGACCT
CGGATCAGGTAGGGATACCCGCTGAACTTAA
>ASV259
TTTCCGTAGGTGAACCTGCGGAAGGATCATTAAGAATTATAAATATTTGTGAAATTTACACAGCAAACAA
TAATTTTATAGTCAAAACAAAAATAATCAAAACTTTTAACAATGGATCTCTTGGTTCTCGTATCGATGAA
GAACGCAGCGNNNNNNNNNNATTGCACTTTGGGGTATCCCCCAAAGTATACTTGTTTGAGCGTTGTTTC
TCTCTTGGAATTGCATTGCTTTTCTAAAATTTCGAATCAAATTCGTTTGAAAAACAACACTATTCAACCTC
AGATCAAGTAGGATTACCCGCTGAACTTAA
>ASV265
TTTCCGTAGGTGAACCTGCGGAAGGATCATTATGAATTAATAATATTTGTGAATTTACCACAGCAAACAA
AAATCATACAATCAAAACAAAAATAATTAAAACTTTTAACAATGGATCTCTTGGTTCTCGTATCGATGAA
GAACGCAGCGNNNNNNNNNNATTGCACTTTGGGGTATCCCCCAAAGTATACTTGTTTGAGCGTTGTTTC
ACTCTTGGAATTGCTTTGCTCTTCTAAAATTTCGAATCAAATTCGTTTGAAAAACAACACTATTCAGCCTC
AGATCAAGTAGGATTACCCGCTGAACTTAA
>ASV268
TTTCCGTAGGTGAACCTGCGGAAGGATCATTACCGAGTGAGGGCCCTTTGGGTCCAACCTCCCACCCGT
GTTTATTTACCTCGTTGCTTCGGCGGGCCCGCCTTAACTGGCCGCCGGGGGGCTCACGCCCCCGGGCCC
GCGCCCGCCGAANNNNNNNNNNGGGCCCGAAAGGCAGCGGCGGCACCGCGTCCGGTCCTCGAGCGTA
TGGGGCTTTGTCACCCGCTCTGTAGGCCCGGCCGGCGCTTGCCGATCAACCCAAATTTTTATCCAGGTTG
ACCTCGGATCAGGTAGGGATACCCGCTGAACTTAA
>ASV269
TTTCCGTAGGTGAACCTGCGGAAGGATCATTACTGTGAATTAACTTCCACACATGCGTGAGCGCACAAA
ACACATAAACCGTGAGTAATTTTAGTCGAAACTTGAAAAAAAAATACAAAACTTTCAACAACGGATCTCT

TGGTTCTCGCANNNNNNNNNNNTTTGAGAAGATGCCAGAGTTGGCCGTGCCACTGGCCCGGCCGAAAAG
AAACGTTGCGGACGAAGCGAACTACATCGGGACGCTTTGGCCGCCGAGCGAAAATATCATTGAGCTCGA
CCTCAGATCAGGTAGGAGTACCCGCTGAACTTAA
>ASV273
TTTCCGTAGGTGAACCTGCGGAAGGATCACTAGTGATTAAATCGAGCGTGTCTTCATTGACCGCTCACCC
TTCTCACCATCCACATACACCTGTGCACTGTTTAGCCTGAGCCGTTTCGGTTCAGGTTATCATTTCATAC
AAACTCTAGTNNNNNNNNNNNTCCACTCAACGTGATAAGTATTTCGTTGAGGACAGTTGCAGCAATGCGG
CTGGCCGGGATAAGAAAGGCATAGTTGTCAGCTTCTAATCGCCCTCGGGCAATTTTTTATGATCTGGCCT
CAAATCAGGTAGGACTACCCGCTGAACTTAA
>ASV281
TTTCCGTAGGTGAACCTGCGGAAGGATCATTAGTGATTGCCTTCTAGGCTTAAACTATATCCACATACAC
CTGTGAACTGTTCTACCACTTGACGCAAGTCGAGTGTTTTTACAAACAATGTGTAATGAACGTCGTTTTA
TTATAACAAANNNNNNNNNNNTCGCCTTAAAAGAGTTAGCAAGTTTGACATTAATGTCTGGTGTAATAAG
TTTCACTGGGTCCATTGTGTTGAAGCGTGCTTCTAATCGTCCGCAAGGACAATTACTTTGACTCTGGCCT
GAAATCAGGTAGGACTACCCGCTGAACTTAA
>ASV282
TTTCCGTAGGTGAACCTGCGGAAGGATCATTAGTGAATTGCTCTCTGAGCGTTAAACTATATCCATCTAC
ACCTGTGAACTGTTGATTGACTTCGGTCAATTACTTTTACAAACATTGTGTAATGAACGTCATGTTATTAT
AACAAAAATNNNNNNNNNNNTGGCTCGCCTTAAAAGAGTTAGCGTGTTTAACTTGTCTATCTGGCGTAAT
AAGTTTCGCTGGTGTCGGCTTGAGAAGTACGCTTCTAATCGTCTTCGGACAATTCTTGAACTCTGGTCTC
AAATCAGGTAGGGCTACCCGCTGAACTTAA
>ASV283
TTTCCGTAGGTGAACCTGCGGAAGGATCATTACTGTGAATATAACTTCCACACATGCGTGAGCGCACAA
AACACATAAACCGTGAGTAATTTTAGTCGAAACTTGAAAAAAAAAATACAAAACTTTCAACAACGGATCT
CTTGGTTCTCGNNNNNNNNNNNAAAGAAAGATCCAGAGCTGGCCGTGCCACTGGCCCGGCCGAAAAGAA
ACGTTGCGGACGAAGCGAACTACATCGGGACGCTTTGGCCGCCGAGCGAAAATATATCATTGAGCTCGA
CCTCAGATCAGGTAGGAGTACCCGCTGAACTTAA
>ASV285
TCTCCGTAGGTGAACCTGCGGAGGGATCATTACAAGTGACCCCGGCTACGGCCGGGATGTTCATAACCC
TTTGTTGTCCGACTCTGTTGCCTCCGGGGCGACCCTGCCTTCGGGCGGGGGCTCCGGGTGGACACTTCA
AACTCTTGCGTANNNNNNNNNNNCGCCGCGTGCCTCAAATCGTCCGGCTGGGTCTTCTGTCCCCTAAGC
GTTGTGGAAACTATTCGCTAAAGGGTGTTCGGGAGGCTACGCCGTAAAACAACCCCATTTCTAAGGTTG
ACCTCGGATCAGGTAGGGATACCCGCTGAACTTAA
>ASV286
TCTCCGTTGGTGAACCAGCGGAGGGATCATTACCGAGTTTACAACTCCCAAACCCAATGTGAACCATAC
CAAACTGTTGCCTCGGCGGGGTCACGCCCCGGGTGCGTCGCAGCCCCGGAACCAGGCGCCCGCCGGAG
GGACCAACCAAACNNNNNNNNNNNGCCCCGAAATACAGTGGCGGTCTCGCCGCAGCCTCTCATGCGCAG
TAGTTTGCACAACTCGCACCGGGAGCGCGGCGCGTCCACGTCCGTAAAACACCCAACTTCTGAAATGTT
GACCTCGGATCAGGTAGGAATACCCGCTGAACTTAA
>ASV287
TTTCCGTAGGTGAACCTGCGGAAGGATCATTACCGAGTTTTCGGGCTTCGGCTCGACTCTCCCACCCTTT
GTGAACGTACCTCTGTTGCTTTGGCGGCTCCGGCCGCCAAAGGACCTCCAAACTCCAGTCAGTAAACGC
AGACGTCTGATNNNNNNNNNNNCGCCTCAAAGACCTCGGCGGTGGCTGTTCAGCCCTCAAGCGTAGTAG
AATACACCTCGCTTTGGAGTGGTTGGCGTCGCCCGCCGGACGAACCTTCTGAACTTTTCTCAAGGTTGAC
CTCGGATCAGGTAGGGATACCCGCTGAACTTAA
>ASV288
TTTCCGTAGGTGAACCTGCGGAAGGATCATTACAGTAGTCGCCCGGGTTGCCGCAAGGCCTCCCGGGTA
ACCTACCACCCTTTGTTTATTACACTTTGTTGCTTTGGCAGGCCTGCCCTCGGGCTGCTGGCTCCGGCCG
GCGAGCGCCTGNNNNNNNNNNNCTCCGGCGGGCCCTAAAGTCAGTGGCGGTGCCGTCCGGCTCCGAGC

GTAGTAATTCTTCTCGCTCTGGAGGTCCGGTCGTGTGCTCGCCAGCAACCCCCAATTTTTTTCAGGTTGA
CCTCGGATCAGGTAGGGATACCCGCTGAACTTAA
>ASV291
TTTCCGTAGGTGAACCTGCGGAAGGATCATTAGCGAAGCTCGGAATGCGTGTTCGGTCTGATGCTGCCC
GGCAACGGGATGTGCTCGGCCGGATGCGTGTCCCTTCTCTATTCCACCCCTTTGTGAACCAAGTGTGCG
AGCCGAAGAGAGNNNNNNNNNNGAATACATTAGCGAAGCCCTTGCGGCCTTGGTGTGATAGTCATCTA
CGCCTCGGTTTAGCGAACATACGGGAATCGCTTCCAACCGTCTTGCAAGAGACAATCACTTCAAACTTG
ACCTCAAATCAGGCGGGACTACCCGCTGAACTTAA
>ASV292
TTTCCGTAGGTGAACCTGCGGAAGGATCATTACCTAGAGTTTGTAGACTTCGGTCTGCTACCTCTTACCC
ATGTCTTTTGAGTACCTTCGTTTCCTCGGCGGGTCCGCCCGCCGATTGGACAACATTCAAACCCTTTGCA
GTTGCAATCANNNNNNNNNNCGTGTAGACTCGCCTTAAAACAATTGGCAGCCGGCGTATTGATTTCGGA
GCGCAGTACATCTCGCGCTTTGCACTCATAACGACGACGTCCAAAAGTACATTTTTTACACTCTTGACCT
CGGATCAGGTAGGGATACCCGCTGAACTTAA
>ASV293
TTTCCGTAGGTGAACCTGCGGAAGGATCATTACCGAGTGTTTGGATGCCCCTTCTCGGGGTGTCCGTCCT
CCCATCCGTGTCTATTTGTACCCTGTTGCTTCGGCGGGCCCGCCCTTCGTGGCCGCCGGGGGGCTTCCC
TGCCCCCGGGCNNNNNNNNNNGGCAGCGGCGGCACCGTGTCCGGTCCGCGAGCGTATGGGAAGCAAC
TTTTTGTCACCCGCTCCTGTAGGTCCGGCCGGCGGCCTGCCCAACCCCAACCTTTTTTTAACCAGGTTGA
CCTCGGATCAGGTAGGGATACCCGCTGAACTTAA
>ASV294
TTTCCGTAGGTGAACCTGCGGAAGGATCATTACCTAGAGTTTGTGGGCTTTGCCCGCTACCTCTTACCCA
TGTCTTTTGAGTACTTACGTTTCCTCGGCGGGTCCGCCCGCCGATTGGACAAAATTAAACCCTTTGCAGT
TGCAATCAGCNNNNNNNNNNGCGTGTAGACTCGCCTCAAAACAATTGGCAGCCGGCGTATTGATTTCG
GAGCGCAGTACATCTCGCGCTTTGCACTCATAACGACGACGTCCAAAAGTACATTTTTACACTCTTGACC
TCGGATCAGGTAGGGATACCCGCTGAACTTAA
>ASV295
TTTCCGTAGGTGAACCTGCGGAAGGATCATTAAGAATTATAATTAATTGTGAAAATTATACAGCAAACAA
TAATTTTATAGTCAAAACAAAAAAAAAAATTAAAACTTTTAACAATGGATCTCTTGGTTCTCGTATCGAT
GAAGAACGCANNNNNNNNNNATTGCACTTTGGGGTATCCCCCAAAGTATACTTGTTTGAGCGTTGTTTC
TCTCTTGGAATTGCATTGCTTTTCTAAAATATTGAATCAAATTCGTTTGAAAAACAACACTATTCAACCTC
AGATCAAGTAGGATTACCCGCTGAACTTAA
>ASV297
TTTCCGTAGGTGAACCTGCGGAAGGATCATTAAAGAGTAAGGGTGCTCAGCGCCCGACCTCCAACCCTT
TGTTGTTAAAACTACCTTGTTGCTTTGGCGGGACCGCTCGGTTCCGAGCCGCTGGGGATTCGTCCCAGG
CGAGTGCCCGCCNNNNNNNNNNGCCTTAAAGACCTCGGCGAGGCCTCACCGGCTTTAGGCGTAGTAGA
ATTTATTCAATTAACGTCTGTCAATGGAGAGGACTTCTGCCGACTGAAACCTTTATTTTTTACAGGTTGA
CCTCGGATCAGGTAGGGATACCCGCTGAACTTAA
>ASV298
TCTCCGTAGGTGAACCTGCGGAGGGATCATTACCGAGTGAGGGCCCTCGCGCCCGACCTCCAACCCTTT
GTCGATTCCTATCTGTTGCCTCGGGGGGCGACCCGGCCGTCCGCGCCCGGGAGTCCCCCAGAGGACCAA
TCAACTCTGCATNNNNNNNNNNCGGGAGACCCCGCGCCCCTAATTCATCGGCTGGACGGTTCGAATCT
CAGCGTTGTGGTCATTCAATTCGCTGGCGAGGACGACCGGACGCGCCGTTAAACCTTTATCACAGGTTG
ACCTCGGATCGGGTAGGGATACCCGCTGAACTTAA
>ASV300
TTTCCGTAGGTGAACCTGCGGAAGGATCATTAAATAATCAATAATTTTGGCTTGTCCATTATTATCTATTT
ACTGTGAACTGTATTATTACTTGACGCTTGAGGGATGCTCCACTGCTATAAGGATAGGCGGTGGGGATGT
TAACCGAGTNNNNNNNNNNTGAAATGTACAAAGGCCTGATCTTGTTTGAATGCCTGAACTTTTTTTTAA
TATAAAGAGAAGCTCTTGCGGTAAACTGTGTTGGGGCCTCCCAAATAATACTCTTTTTAAATTTGATCTG
AAATCAGGCGGGATTACCCGCTGAACTTAA

>ASV305
TTTCCGTAGGTGAACCTGCGGAAGGATCATTAATGATTGGCCTAACGGCCTTATCAACATAATCCCTCAC
CTCTGTGAACCGTTGACCTCCGGGTCTACATAACAAACATCAGTGTAAAGAACGTCTTTATATTTTAACA
AAATAAAACTNNNNNNNNNNGCCTTAAATGACTCAGTGGGATCTTCAGCATCCGTGGCAGACGTAATA
AGTTTCGTCTCGTCCCTTGCTGTGAGGACCGCTCATAACCTGCCATCGCGCACCACTTTTGACTCTGACC
TCAAATCAGGTAGGACTACCCGCTGAACTTAA
>ASV309
TTTCCGTAGGTGAACCTGCGGAAGGATCATTATGAATTAATAATATTTGTGAAATTTCAACAAACAACAT
CAATTTTATAGTCAAAACAAAAAAAAATTAAAACTTTTAACAATGGATCTCTTGGTTCTCGTATCGATGA
AGAACGCAGCNNNNNNNNNNATTGCACTTTGGGGTATCCCCCAAAGTATACTTGTTTGAGCGTTGTTTC
TCTCTTGGAATTGCATTGCTTTTCTAAAATTTCGAATCAAATTCGTTTGAAAAACAACACTATTCAACCTC
AGATCAAGTAGGATTACCCGCTGAACTTAA
>ASV311
TTTCCGTAGGTGAACCTGCGGAAGGATCATTACCGAGTGTAGGGTTCCTAGCGAGCCCAACCTCCCACC
CGTGTTTACTGTAACCTTAGTTGCTTCGGCGGGCCCGCCTTTAAGGCCGCCGGGGGGCATCAGCCCCCG
GGCCCGCGCCCGNNNNNNNNNNCCCCAAAGGCAGCGGCGGCACCGCGTCCGATCCTCGAGCGTATGG
GGCTTTGTCACCCGCTCTGTAGGCCCGGCCGGCGCTTGCCGAACGCAAAACAACCATTCTTTCCAGGTT
GACCTCGGATCAGGTAGGGATACCCGCTGAACTTAA
>ASV312
TTTCCGTAGGTGAACCTGCGGAAGGATCATTAAAAATATTATTACACCCTTTTAGGCACAAACTCTAAAT
CTTAACCGTCAATAATTCTTTAAAAAAACTTTCAACAACGGATCTCTTGGTTCTCGCATCGATGAAGAAC
GCAGCGAATTNNNNNNNNNNCCGGGGTATTCCCCAGGGCATGCGTGGGTGAGCGATATTTACTCTCAA
ACCTCCGGTTTGGTCCTGCTTCGGCCTAATATCAACGGCGCTAGAATAAGTTTTAGCCCCATTCTTCTTC
CTCACCCTCGTAAGACTACCCGCTGAACTTAA
>ASV313
TTTCCGTAGGTGAACCTGCGGAAGGATCATTATGAATTAAAAATATTTGTGAATTTACCACAACAAACAA
AAATACTATAGTCAAAACAAAAATAATTAAAACTTTTAACAATGGATCTCTTGGTTCTCGTATCGATGAA
GAACGCAGCGNNNNNNNNNNATTGCACTTTGGGGTATCCCCCAAAGTATACTTGTTTGAGCGTTGTTTC
TCTCTTGGAATTGCTTTGCTCTTCTAAAATTTCGAATCAAATTCGTTTGAAAAACAACACTATTCAACCTC
AGATCAAGTAGGATTACCCGCTGAACTTAA
>ASV314
TTTCCGTAGGTGAACCTGCGGAAGGATCATTACCGAGTGTAGGGTTCCTAGCGAGCCCAACCTCCCACC
CGTGTTTACTGTACCTTAGTTGCTTCGGCGGGCCCGCCATTCATGGCCGCCGGGGGCTCTCAGCCCCGG
GCCCGCGCCCGCNNNNNNNNNNGCCCGAAAGGCAGCGGCGGCACCGCGTCCGGTCCTCGAGCGTATG
GGGCTTCGTCACCCGCTCTGTAGGCCCGGCCGGCGCCCGCCGGCGAACACCATCAATCTTAACCAGGTT
GACCTCGGATCAGGTAGGGATACCCGCTGAACTTAA
>ASV316
TTTCCGTAGGTGAACCTGCGGAAGGATCATTAAATAATCAATAATTTTGGCTTGTCCATTATTATCTATTT
ACTGTGAACTGTATTATTACTTGACGCTTGAGGGATGCTCCACTGCTATAAGGATAGGCGGTGGGGATGT
TAACCGAGTNNNNNNNNNNTGAAATGTACAAAGGCCTGATCTTGTTTGAATGCCTGAACTTTTTTTTAA
TATAAAGAGAAGCTCTTGCGGTAAACTGTGCTGGGGCCTCCCAAATAATACTCTTTTTAAATTTGATCTG
AAATCAGGCGGGATTACCCGCTGAACTTAA
>ASV317
TTTCCGTAGGTGAACCTGTTTGAGCGTCGTTTCTCCCTCAAACCGCTGGGTTTGGTGTTGAGCAATACGA
CTTGGGTTTGCTTGAAAGACGGTAGTGGTAAGGCGGGATCGCTTTGACAATGGCTTAGGTCTAACCAAA
AACATTGCTTGNNNNNNNNNNTGGGTTTGCTTGAAAGACGGTAGTGGTAAGGCGGGATCGCTTTGACA
ATGGCTTAGGTCTAACCAAAAACATTGCTTGCGGCGGTAACGTCTACCACGTATATCTTCAAACTTTGAC
CTCAAATCAGGTAGGACTACCCGCTGAACTTAA
>ASV318

TTTCCGTAGGTGAACCTGCGGAAGGATCATTAATGTGTGTCTGTGCGGCTAGGTGCCCCTAAACAAGGC
CCTGCCGCGCACTCCCACCCTTGTCTACCTTACCTGTTGCTTCGGCGGGCCTGCGGGTTCTCGCGAGCCT
GCTGCCGGAGGNNNNNNNNNNGCCCGAAAGGCAGTGACGGCGTCGTGTTCCGGTGCCCGAGCGTATG
GGGCTTTGTCTTTCGCTCTAGTGGCCTGGCCGACTGTCCGGTCTAACCATCATTTACTTCTAGTGGTTGA
CCTCGGATCAGGTAGGGTTACCCGCTGAACTTAA
>ASV320
CTCCCTCAAACCGCTGGGTTTGGTGTTGAGCAATACGACTTGGGTTTGCTTGAAAGACGGTAGTGGTAA
GGCGGGATCGCTTTGACAATGGCTTAGGTCTAACCAAAAACATTGCTTGCGGCGGTAACGTCTACCACG
TATATCTTCAAANNNNNNNNNNNTGGGTTTGCTTGAAAGACGGTAGTGGTAAGGCGGGATCGCTTTGACA
ATGGCTTAGGTCTAACCAAAAACATTGCTTGCGGCGGTAACGTCTACCACGTATATCTTCAAACTTTGAC
CTCAAATCAGGTAGGACTACCCGCTGAACTTAA
>ASV321
TTTCCGTAGGTGAACCTGCGGAAGGATCATTACCGAGTGCGGGCTGCCTCCGGGCGCCCAACCTCCCAC
CCGTGACTACCTAACACTGTTGCTTCGGCGGGGAGCCCTCTCGGGGGCGAGCCGCCGGGGACTACTGAA
CTTCATGCCTGANNNNNNNNNNCCCGAAAGGCAGCGGCGGCACCGTGTCCGGTCCTCGAGCGTATGGG
GCTTTGTCACCCGCTCGATTAGGGCCGGCCGGGCGCCAGCCGACGTCTCCAACCATTTCTTCAGGTTG
ACCTCGGATCAGGTAGGGATACCCGCTGAACTTAA
>ASV322
TTTCCGTAGGTGAACCTGCGGAAGGATCATTCCCAATTTTTTTTTTCTCTCTTCATTGAGAGGAAAGAAT
TTGGTATTCACCCAGTCTATTGCAACGATTCCTGGGTTAACAAAGAATGGATTTTCAATTAAAAACATTT
TTTTTTAATTNNNNNNNNNNAAATGAGTCCATAGGATTAAAAATCAATTGAGGTTTTTTTTTTCCTTTGC
ATCAAATTTTTTTCAATTAGAAAAAAAGCAATTGGGAAAAAAGGATCCAATTCTTCAAACTCGTTGTCTG
AAATCAAGTAGGATTACCCGCTGAACTTAA
>ASV324
TCTCCGTAGGTGAACCTGCGGAGGGATCATTACCCAAATATGAAACGCAGACTGGGCACCCTCGAGGAG
CGATTCGTCGTCCCCCTCCTGGGCCGGTCTGACGCCATATTCACCCATGTCTTTTGCGTACTACTTGTTT
CCTTGGCGGGTNNNNNNNNNNCCCCGAGACTCGCCTTAAAATCATTGGCAGCCGGCCTACTGGTTTCG
GAGCGCAGCACATTTTTTGCGCTTTGGTCTAGTGGTCCAGCGTCCATGAAGCGAATATTTTCAATTTGAC
CTCGGATCAGGTAGGGATACCCGCTGAACTTAA
>ASV327
TTTCTGTAGGTGAACCTGCAGAAGGATCATTAGTGAAGATTTGGGCAGGCCATACGGACGCCAAAAAGT
GTCCCTGGCCGCCTACACCCACTATACATCCACAAACCCGTGTGCACTGTCTTGGAGAAAGGCTTCAGA
GAAGTTTTTTGTNNNNNNNNNNTTTCTCCTGGCATGGCATGATACGTCATTTGCTATGTCGCCTAAAGG
AGGAATGTTTGGTTGTGTCTGCGTGTGCTTCGAATTTGCCTCTGTGGCACATCCCAATTTCACTTCTGGT
CTCAAATCAGGTAGGATCACCCGCTGAACTTAA
>ASV328
TTTCCGTAGGTGAACCTGCGGAAGGATCATTACCGAGTGCGGGCTGCCTCCGGGCGCCCAACCTCCCAC
CCGTGACTACCTAACACTGTTGCTTCGGCGGGGAGCCCTCTCGGGGGCGAGCCGCCGGGGACTACTGAA
CTTCATGCCTGANNNNNNNNNNGGTCCGAAAGGCAGCGGCGGCACCGAGTCCGGTCCTCGAGCGTATG
GGGCTTTGTCACCCGCTCTGTAGGCCCGGCCGGCGCCAGCCGACAACCAATCATCCTTTTTTCAGGTTG
ACCTCGGATCAGGTAGGGATACCCGCTGAACTTAA
>ASV329
TTTCCGTAGGTGAACCTGCGGAAGGATCATTACTGATTTGCTTAATTGCACCACATGTGTTTTTCTTTGA
AACAAACTTGCTTTGGCGGTGGGCCCAGCCTGCCGCCAGAGGTCTAAACTTACAACCAATTTTTTATCAA
CTTGTCACACNNNNNNNNNNACATGTGTTTTTCTTTGAAACAAACTTGCTTTGGCGGTGGGCCCAGCCT
GCCGCCAGAGGTCTAAACTTACAACCAATTTTTTATCAACTTGTCACACCAGATTATTACTAATAGTCAA
AACTTTCAACAACGGATCTCTTGGTACTTAA
>ASV330
TCTCCGTTGGTGAACCAGCGGAGGGATCATTACCGAGTTTACAACTCCCAAACCCATGTGAACATACCT
ACTGTTGCTTCGGCGGGATTGCCCCGGGCACCTCGTGTGCCCCGGATCAGGCGCCCGCCTAGGAAACTT

AACTCTTGTTTTNNNNNNNNNNNCGGCCGGCCCCTAAATCTAGTGGCGGACCCGTCGTGGCCTCCTCTGC
GAAGTAGTAATATTCCGCATCGGACAGCGACGAGCCCCTGCCGTTAAACCCCCAACTTTCCAAGGTTGA
CCTCAGATCAGGTAGGAATACCCGCTGAACTTAA
>ASV331
TCTCCGTAGGTGAACCTGCGGAGGGATCATTACACAATAACCAGGCGGGCTGGACACCCCCCGCTGGGC
ACTGCTTCACGGCGTGCGCGGCGGGGCCGGCCCTGCTGAATTATTCACCCGTGTCTTTTGCGTACTTCTT
GTTTCCTGGGTNNNNNNNNNNNTGGAGACTCGCCTTAAAGTAATTGGCAGCCGGCCTACTGGTTTCGGA
GCGCAGCACAAGTCGCACTCTCTATCAGCAAAGGTCTAGCATCCATTAAGCCTTTTTTTCAACTTTTGAC
CTCGGATCAGGTAGGGATACCCGCTGAACTTAA
>ASV332
TTTCCGTAGGTGAACCTGCGGAAGGATCATTACTGATAGTTTTAGTTTGTTGCCTGCGCTTAATTGCGCG
GTGACAAGCAAACACCTTACACACTGTGTTTTTGTTTTATTGAAAACTTGCTTTGGTTTGGCGCAAGCTG
GGCCAAAGACNNNNNNNNNNNTGTACTGGATAGTACGAACTGGTTATTCAATGTATTAGGTTTATCCAAC
TCGTTGAAGACTGGGGTAGTAAATTTCTAGTAATTGGCTCGGCCTTATAATAACAAACTAAGTTTGACCT
CAAATCAGGTAAGAATACCCGCTGAACTTAA
>ASV333
TTTCCGTAGGTGAACCTGCGGAAGGATCATTAATGATTGAACGTCTGTCGAGCTTGCTCACAGGCACATC
ATATCCATAACACCTGTGCACTTGTCGGATGGCTTAGTGAAGACCGCAAGGTTGGATCTATCCATCTACT
TTACATAACANNNNNNNNNNNTGGTTTGGCTTGGCGTAATAAGTATTTCGCTAAGGACATCTTCGGATGG
CCGCGTTGCAGGACTAAAGACCGCTTTCTAATCCATTGATCTTACGATTAATATTCTTGACATCTGGCCT
CAAATCAGGTAGGACTACCCGCTGAACTTAA
>ASV334
TTTCCGTAGGTGAACCTGCGGAAGGATCATTAGTGAATATTAGCGCATCTACTTGTAGAGCGTGACCTCC
ACTTTCTAACTCTGTGCATTTATTGGCGGAAGAGACTTGAGCAATCGAGTCTTCTTCTCGCGGCTCATTT
TATAACACTANNNNNNNNNNNTTCGAACTCGGATTGACTCAGTGTAATAGACTATTCGCTGAGGACACGC
TCTTGTAGTGTGGCCGAATGAGATCTCAGTAGAAGCTTCCAACTACTTTAGTCAACTTTAGATTAGACCT
CAGATCAGGCAGGATTACCCGCTGAACTTAA
>ASV335
TTTCCGTAGGTGAACCTGCGGAAGGATCATTACCGAGTGAGGGCCCTCTGGGTCCAACCTCCCACCCGT
GTTTATTTTACCTTGTTGCTTCGGCGAGCCTGCCTTTTGGCTGCCGGGGGACATCTGTCCCCGGGTCCGC
GCTCGCCGAAGNNNNNNNNNNNCGGGCCCGAAAGGCAGCGGCGGCACCGCGTCCGGTCCTCAAGCGTA
TGGGGCTTTGTCACCCGCTTTGTAGGACTGGCCGGCGCCTGCCGATCAACCAAACTTTTTTCCAGGTTGA
CCTCGGATCAGGTAGGGATACCCGCTGAACTTAA
>ASV338
TTTCCGTAGGTGAACCTGCGGAAGGATCATTATGAATTAATAATATTTGTGAAATTTCAACAAACAACAA
CAATCATACAATCAATAATTAAAAAATTAAAACTTTTAACAATGGATCTCTTGGTTCTCGTATCGATGAA
GAACACAGCGNNNNNNNNNNNATTGCACTTTGGGGTATCCCCCAAAGTATACTTGTTTGAGCGTTGTTTC
TCTCTTGGAATTGCTTTGCTCTTCTAAAATTTCGAATCAAATTCGTTTGAAAAACAACACTATTCAACCTC
AGATCAAGTAGGATTACCCGCTGAACTTAA
>ASV340
TTTCCGTAGGTGAACCTGCGGAAGGATCATTACCTAGAGTTTGTAGACTTCGGTCTGCTACCTCTTACCC
ATGTCTTTTGAGTACCTTCGTTTCCTCGGCGGGTCCGCCCGCCGATTGGACAACATTCAAACCCTTTGCA
GTTGCAATCANNNNNNNNNNNGCGTGTAGACTCGCCTTAAAACAATTGGCAGCCGGCGTATTGATTTCG
GAGCGCAGTACATCTCGCGCTTTGCACTCATAACGACGACGTCCAAAAGTACATTTTTACACTCTTGACC
TCGGATCAGGTAGGGATACCCGCTGAACTTAA
>ASV341
TTTCCGTAGGTGAACCTGCGGAAGGATCACTAGTGATTAAATCGAGAGCGTCTTCATTGACCTCTCACCC
TTCACATCCACATACACCTGTGCACCGTTTGGCTCTTATAAAAGACGCAAGTCTGCAATGAGAGTCATCA
ATTTTATACANNNNNNNNNNNGTGATAAGTATTTCGCCGAGGACATACGCAAGTATGGCCGAGATAAAG

GAAGTCTTTAGATCCGCTTCTAATTCTTAGATAGAGCTTGCTCTACTAAACCCCATTTTATGATCTGGCC
TCAAATCAGGTAGGACTACCCGCTGAACTTAA
>ASV344
TTTCTGTAGGTGAACCTGCAGAAGGATCATTAGTGAAGATTTGGGCAGGCCATACGGACGCCAAAAAAG
TGTCCCTGGCCGCCTACACCCACTATACATCCACAAACCCGTGTGCACTGTCTTGGAGAAAGGCTTCTTG
AGAAGTTTTGTNNNNNNNNNNTTTCTCCAGGCATGGCATGATACGTCATTTGCTATGTCGCCCAATGG
AGGAATGTTTGGTTGTGTCTGCGTGTGCTTCGAACTTGCCTCTGTGCAAAATCCCCTTTCACTTCTGGTC
TCAAATCAGGTAGGATCACCCGCTGAACTTAA
>ASV347
TCTCCGTTGGTGAACCAGCGGAGGGATCATTACCGAGTTTACAAACTCCCAAACCCTTTGTGAACCTTAC
CTATCGTTGCTTCGGCGGGATCGCCCCGGGCCGGCCCCTTCGCGGGGGCCCTCCGGAGCCAGGCGCCC
GCCGGAGAACCCNNNNNNNNNNCCGGCTCCGAAATCTAGTGGCGGTCTCGCTGTAGTCCTCCTCTGCG
TAGTAGCACAACCTCGCAGTTGGAACGCGGCGGTGGCCATGCCGTTAAACACCCCACTTCTGAAAGTTG
ACCTCGGATCAGGTAGGAATACCCGCTGAACTTAA
>ASV348
TTTCCGTAGGTGAACCTGCGGAAGGATCATTACCTAGAGTTGTGGGCTTTGCCTGCTATCTCTTACCCAT
GTCTTTTGAGTACTTACGTTTCCTCGGTGGGTTCGCCCGCCGATTGGACAATTTAAACCCTTTGCAGTTG
CAATCAGCGTNNNNNNNNNNGCGTGTAGACTCGCCTTAAAACAATTGGCAGCCGGCGTATTGATTTCG
GAGCGCAGTACATCTCGCGCTTTGCACTCATAACGACGACGTCCAAAAGTACTTTTTTACACTCTTGACC
TCGGATCAGGTAGGGATACCCGCTGAACTTAA
>ASV351
TCTCCGTTGGTGAACCAGCGGAGGGATCATTACCGAGTTTACAACTCCCAAACCCCTGTGAACATACCA
ATTGTTGCCTCGGCGGATCAGCCCGCTCCCGGTAAAACGGGACGGCCCGCCAGAGGACCCCTAAACTCT
GTTTCTATATGTNNNNNNNNNNGCCGGCCCCGAAATCTAGTGGCGGTCTCGCTGCAGCTTCCATTGCGT
AGTAGTAAAACCCTCGCAACTGGTACGCGGCGCGGCCAAGCCGTTAAACCCCCAACTTCTGAATGTTGA
CCTCGGATCAGGTAGGAATACCCGCTGAACTTAA
>ASV353
TTTCCGTAGGTGAACCTGCGGAAGGATCATTACCGAGTGAGGGCCCTTTGGGTCCAACCTCCCACCCGT
GTTTATTTTACCTTGTTGCTTCGGCGGGCCCGCCTTTACTGGCCGCCGGGGGGCTCACGCCCCCGGGCC
CGCGCCCGCCGANNNNNNNNNNCGGGCCCGAAAGGCAGCGGCGGCACCGCGTCCGGTCCTCGAGCGT
ATGGGGCTTTGTCACCCGCTCTGTAGGACTGGCCGGCGCCTGCCGATCAACCAAACTTTTTTCCAGGTTG
ACCTCGGATCAGGTAGGGATACCCGCTGAACTTAA
>ASV354
TTTCCGTAGGTGAACCTGCGGAAGGATCATTACTGTGAATATAACTTCCACACATGCGTGAGCGCACAA
AACACATAAACCGTGAGTAATTTTAGTCGAAACTTGAAAAAAAAATACAAAACTTTCAACAACGGATCTC
TTGGTTCTCGCNNNNNNNNNNTTAAAGAAAGATCCAGAGCTGGCCGTGCCACTGGCCCGGCCGAAAAG
AAACGTTGCGGACGAAGCGAACTACATCGGGACGCTTTGGCCGCCGAGCGAAAATATCATTGAGCTCGA
CCTCAGATCAGGTAGGAGTACCCGCTGAACTTAA
>ASV355
TTTCCGTAGGTGAACCTGCGGAAGGATCATTACTGATTTGCTTAATTGCACCACATGTGTTTTTCTTTGA
AACAAACTTGCTTTGGCGGTGGGCCCAGCCTGCCGCCAGAGGTCTAAACTTACAACCAATTTTTTATCAA
CTTGTCACACNNNNNNNNNNTTTGAAACAAACTTGCTTTGGCGGTGGGCCCAGCCTGCCGCCAGAGGT
CTAAACTTACAACCAATTTTTTATCAACTTGTCACACCAGATTATTACTTAATAGTCAAAACTTTCAACAA
CGGATCAAGTAGGATTACCCGCTGAACTTAA
>ASV356
TTTCCGTAGGTGAACCTGCGGAAGGATCATTACTGTGATTTTAACATCTTTACACTGCGTGAGCGCACAA
CAACACCTAAACATGAATACTTAATAGTCAAAACTTTCAACAACGGATCTCTTGGTTCTCGCATCGATGA
AGAACGCAGCNNNNNNNNNNTCGGGTTTGGTGTTGAGCGATACGCTGGGTTTGCTTGAAAGAAAGGCG
GAGTATAAACTAATGGATAGGTTTTTTCCACTCATTGGTACAAACTCCAAAACTTCTTCCAAATTCGACC
TCAAATCAGGTAGGACTACCCGCTGAACTTAA

>ASV357
ATTGCAGATATTCGTGAATCATCGAATCTTTGAACGCACATTGCGCCCTCTGGTATTCCGGAGGGCATGC
CTGTTTGAGCGTCGTTTCTCCCTCAAACCGCTGGGTTTGGTGTTGAGCAATACGACTTGGGTTTGCTTGA
AAGACGGTAGNNNNNNNNNNTGGGTTTGCTTGAAAGACGGTAGTGGTAAGGCGGGATCGCTTTGACAA
TGGCTTAGGTCTAACCAAAAACATTGCTTGCGGCGGTAACGTCCACCACGTATATCTTCAAACTTTGACC
TCAAATCAGGTAGGACTACCCGCTGAACTTAA
>ASV358
TTTCCGTAGGTGAACCTGCGGAAGGATCATTACCGAGTGCGGGCTGCCTCCGGGCGCCCAACCTCCCAC
CCGTGACTACCTAACACTGTTGCTTCGGCGGGGAGCCCCCAGGGGCGAGCCGCCGGGGACCACTGAAC
TTCATGCCTGAGNNNNNNNNNNGGGCCCGAAAGGCAGCGGCGGCACCGCGTCCGGTCCTCGAGCGTAT
GGGGCTTTGTCACCCGCTCTGTAGGCCCGGCCGGCGCTTGCCGATCAACCCAAATTTTTATCCAGGTTG
ACCTCGGATCAGGTAGGGATACCCGCTGAACTTAA
